# 12.6 μm-Thick Asymmetric Composite Electrolyte with Superior Interfacial Stability for Solid-State Lithium-Metal Batteries

**DOI:** 10.1007/s40820-024-01389-2

**Published:** 2024-04-26

**Authors:** Zheng Zhang, Jingren Gou, Kaixuan Cui, Xin Zhang, Yujian Yao, Suqing Wang, Haihui Wang

**Affiliations:** 1 Beijing Key Laboratory for Membrane Materials and Engineering, Department of Chemical Engineering, Tsinghua University, Beijing, 100084 People’s Republic of China; 2 School of Chemistry and Chemical Engineering, South China University of Technology, Guangzhou, 510000 People’s Republic of China

**Keywords:** Solid-state lithium metal batteries, Composite solid-state electrolyte, Ultrathin asymmetric structure, Pouch cells

## Abstract

Solid-state lithium metal batteries (SSLMBs) show great promise in terms of high-energy–density and high-safety performance. However, there is an urgent need to address the compatibility of electrolytes with high-voltage cathodes/Li anodes, and to minimize the electrolyte thickness to achieve high-energy–density of SSLMBs. Herein, we develop an ultrathin (12.6 µm) asymmetric composite solid-state electrolyte with ultralight areal density (1.69 mg cm^−2^) for SSLMBs. The electrolyte combining a garnet (LLZO) layer and a metal organic framework (MOF) layer, which are fabricated on both sides of the polyethylene (PE) separator separately by tape casting. The PE separator endows the electrolyte with flexibility and excellent mechanical properties. The LLZO layer on the cathode side ensures high chemical stability at high voltage. The MOF layer on the anode side achieves a stable electric field and uniform Li flux, thus promoting uniform Li^+^ deposition. Thanks to the well-designed structure, the Li symmetric battery exhibits an ultralong cycle life (5000 h), and high-voltage SSLMBs achieve stable cycle performance. The assembled pouch cells provided a gravimetric/volume energy density of 344.0 Wh kg^−1^/773.1 Wh L^−1^. This simple operation allows for large-scale preparation, and the design concept of ultrathin asymmetric structure also reveals the future development direction of SSLMBs.

**Supplementary Information:**

The online version contains supplementary material available at 10.1007/s40820-024-01389-2.

## Highlights

- Ultra-thin asymmetric composite solid-state electrolytes with ultralight areal density were designed for solid-state lithium metal batteries, achieving interfacial stability to Li metal and high-voltage cathode.
- The improved mechanical properties of the electrolyte contribute to the inhibition of Li dendrite growth was demonstrated by both experimental and theoretical simulations.
- The assembled pouch cell exhibited a high gravimetric/volume energy density of 344.0 Wh kg^−1^/773.1 Wh L^−1^.

**Supplementary Information:**

The online version contains supplementary material available at 10.1007/s40820-024-01389-2.

## Graphical abstract

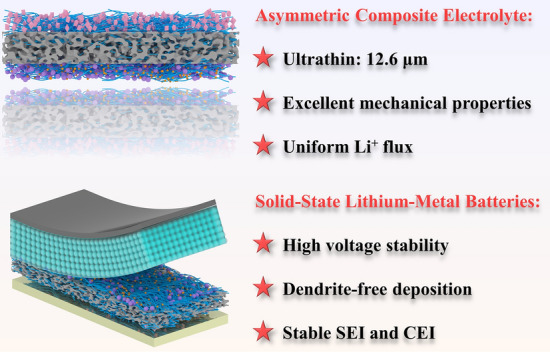

## Introduction

Due to the limitations of traditional lithium-ion batteries in terms of energy density and safety, solid-state batteries with higher energy density and higher safety have become a hot topic in recent years, especially in the field of electric vehicles [1–3]. The composite solid-state electrolytes (CSEs) consisting of polymer electrolytes and inorganic fillers, which inherit the advantages of both organic and inorganic phases, are considered as one of the most promising candidates for future solid-state lithium metal batteries (SSLMBs) [4–6]. However, the most existing CSEs have problems such as over-thick electrolytes and poor interfacial properties of SSLMBs, which seriously hinder the development of SSLMBs and fail to meet the future market demand.

In order to solve the above-mentioned problems, a large number of researches focus on the electrolyte structure design. Specially structured electrolytes not only optimize the Li^+^ transport path, but also help to solve the interfacial problem of SSLMBs, which is important for improving the energy density of battery. There are two interfaces in SSLMBs, including the electrolyte/anode interface (anode side) and the electrolyte/cathode interface (cathode side) [7, 8]. On the anode side, the uncontrollable growth of Li dendrites leads to a short circuit of the battery, further causing safety incidents. On the cathode side, a wide electrochemical window is essential to construct a stable cathode interface, which is of great significance for matching the electrolyte with the high-voltage cathode to achieve high energy density of SSLMBs. At the current stage, it is extremely challenging to find a single electrolyte that takes into account both the cathode and anode interfaces, so it is particularly important to construct an asymmetric electrolyte. In asymmetric structures, each electrolyte layer exerts its respective advantages, contributing to the excellent electrochemical performance of SSLMBs [9]. For example, Zhang et al. designed a double-layer electrolyte consisting of a high-voltage layer (PAN/IL) and a lithium-compatible layer (PVDF-HFP/UiO-66-SO_3_Li) [10], where the high-voltage layer achieves electrochemical stability and low interfacial resistance, and the Li-compatibility layer uniformly distributes the potential and Li^+^ gradients in the electrolyte, resulting in a stable Li^+^ flux. However, the mechanical strength of those CSEs is low due to the lack of robust supports, making it difficult to achieve longer Li plating/stripping cycles.

In addition, the thickness of the electrolyte is also one of the important factors affecting the energy density of SSLMBs. Ultrathin electrolytes have the following advantages [11, 12], on the one hand, the mass/thickness of the electrolyte is reduced, which can improve the gravimetric/volume energy density of SSLMBs. On the other hand, the ultrathin electrolyte helps to reduce the ohmic resistance of the battery, which is beneficial to improve the battery rate performance. However, the dilemma between minimizing the electrolyte thickness and maintaining the mechanical strength still needs to be balanced. As the lack of support in the electrolyte makes it difficult to obtain ultrathin electrolytes by simply mixing fillers with polymers, which requires the introduction of robust and reliable supports in the polymer matrix. There have been several reports on the use of polyimide (PI) [14], polyethylene (PE) separators [15], polytetrafluoroethylene (PEO) [16] and polyacrylonitrile nanofiber [17] membranes as supports for the fabrication of ultrathin electrolytes, which have a shorter ion transport path significantly reducing the ionic transport impedance of the electrolyte and achieve excellent electrochemical performance. However, the presence of only a polyethyleneoxide polymer matrix in the system that make it difficult to match with a high-voltage cathode, which limits the further increase in the energy density of SSLMBs.

Herein, we report an ultrathin asymmetric electrolyte for stabilizing SSLMBs interfaces. Using an ultrathin PE separator as flexible supports, the LLZO and metal organic framework (MOF) layers can be easily coated on both sides of the PE separator by tape casting, and this facile approach is well compatible with the roll-to-roll manufacturing process. Specifically, the LLZO layer consisting of PEO, LiTFSI, LLZO, and succinonitrile (SN), the MOF layer consists of PEO, LiTFSI, and Li-IL@MOFs. The PE separator allows the polymer matrix to penetrate to form a continuous conduction path, minimizing the electrolyte thickness (12.6 µm) and providing excellent mechanical properties for the electrolyte. In addition, the ultrathin electrolyte achieves an ultralight areal density (1.69 mg cm^−2^). On the anode side, the MOF layer in contact with Li metal acts as a "securer" to achieve a more uniform electric field and Li flux, and the interfacial chemical analysis revealed the formation of a multicomponent solid electrolyte interface (SEI). On the cathode side, LLZO and SN provide interfacial stability at high-voltage, leading to excellent cycling stability of batteries when assembled with high-voltage cathodes. The NCM811/Li pouch cell using the ultrathin asymmetric electrolyte achieves a high gravimetric/volume energy density of 344.0 Wh kg^−1^/773.1 Wh L^−1^. The design concepts proposed in this study provide guidance for the development of high-performance SSLMBs.

## Experimental Section

The LLZO layer precursor solution was obtained by dissolving 0.25 g PEO, 0.15 g LiTFSI, 0.12 g LLZO, and 0.104 g SN in anhydrous acetonitrile. The MOF precursor solution was obtained by dissolving 0.25 g PEO, 0.15 g LiTFSI, and 0.12 g Li-IL@MOFs in anhydrous acetonitrile. Ultrathin asymmetric electrolytes (PLM) were prepared by coating LLZO layer and MOF layer on both sides of the PE separator by tape casting, respectively. To illustrate the necessity of asymmetric structure design, LLZO symmetric electrolyte (PLS) and MOF symmetric electrolyte (PMI), PEO/SN symmetric electrolyte (PEO/SN-PE) and PEO symmetric electrolyte (PEO-PE) were coated on both sides of the PE separator for electrochemical performance testing. Among them, precursor solutions without LLZO and MOFs were used as precursor solutions for PEO/SN-PE and PEO-PE electrolytes, respectively. In addition, PEO electrolytes were prepared without fillers in the polymer matrix, and the EO:Li was controlled at 15:1 in order to obtain free-standing PEO films. These electrolytes were placed in a vacuum oven at 40 °C for 24 h to further remove anhydrous acetonitrile. Detailed experimental details are listed in the Supplementary Material.

## Results and Discussion

### Structural Characterizations

**Fig. 1:**
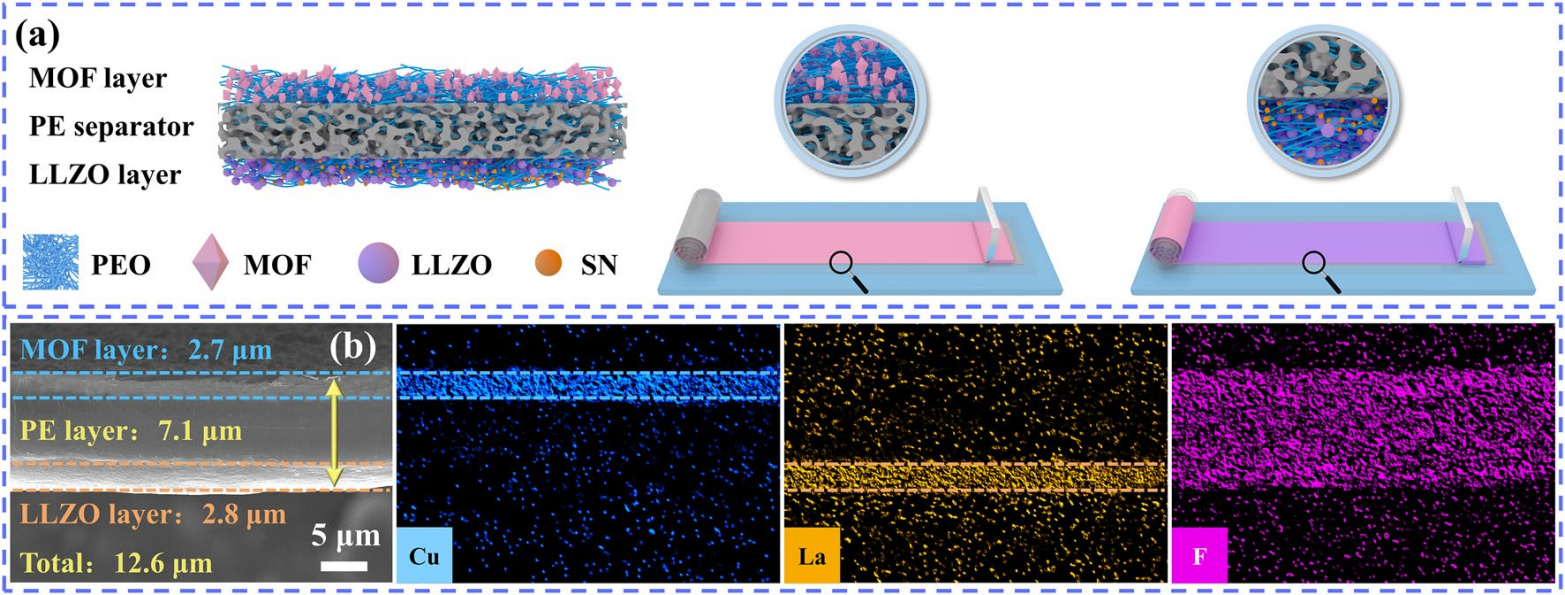
**a** Fabrication process of the ultrathin asymmetric PLM electrolyte using tape casting. **b** Cross-sectional SEM images of PLM and EDS mapping

Figure 1a shows the fabrication process of an ultrathin asymmetric composite electrolyte (denoted as PLM), which was obtained by tape casting the LLZO and MOF layers onto both sides of the PE separator, respectively. Specifically, LLZO particles were prepared by solid-phase sintering (Fig. S1), and the particle size distribution curves showed that the LLZO particle size was mainly concentrated at 410 nm. The X-ray diffraction (XRD) patterns indicates that the nanometer-sized LLZO exhibits a cubic garnet structure and a relatively pure phase [17]. The LLZO layers were prepared by dispersing LLZO/SN in PEO/LiTFSI matrix, in which LLZO nanoparticles can provide more conduction paths for ion transport [18], and the incorporation of SN can improve the high-voltage stability of the polymer matrix [19]. MOFs nanoparticles were prepared by solution method (Fig. S2), scanning electron microscopy (SEM) images showed that MOFs exhibited an ortho-octahedral structure with an average particle size of 610 nm, and XRD pattern indicated that MOFs showed a polycrystalline structure. To improve the electrolyte/Li metal interfacial performance [20], MOFs were compounded with Li-containing ionic liquids (Li-IL@MOFs, Figs. S2–S4). The CSEs prepared by adding the Li-IL@MOFs into the PEO/LiTFSI matrix are represented as MOF layers. Finally, the PE separator was used as a flexible support framework, thanks to its porous structure (Fig. S5) which facilitates the penetration of polymer matrix solution forming a continuous conduction path.

The surface morphology of the PLM electrolyte was observed by SEM and compared to the smooth and flat surface of the PEO electrolyte (Fig. S6), MOFs and LLZO were well dispersed in the polymer matrix and covered on both sides of the PE separator (Fig. S7). From the cross-sectional images, the thickness of the PLM electrolyte is as low as 12.6 μm (Fig. 1b), where Cu and La are enriched on both sides of the electrolyte corresponding to the MOF layer (2.7 μm) and the LLZO layer (2.8 μm), respectively. In addition, the F element from LiTFSI penetrates throughout the electrolyte, indicating that the electrolyte solution can effectively penetrate the PE framework. The lack of a robust support makes it difficult to achieve ultra-thin freestanding PEO electrolyte films, which is about 120 μm (Fig. S8). More importantly, it can be prepared on a large scale by the tape casting method, which is easily incorporated into the roll-to-roll manufacturing process. Figure S9 shows that the PLM electrolyte have low thickness and good flexibility, which is expected to be used in flexible electronic devices.

**Fig. 2:**
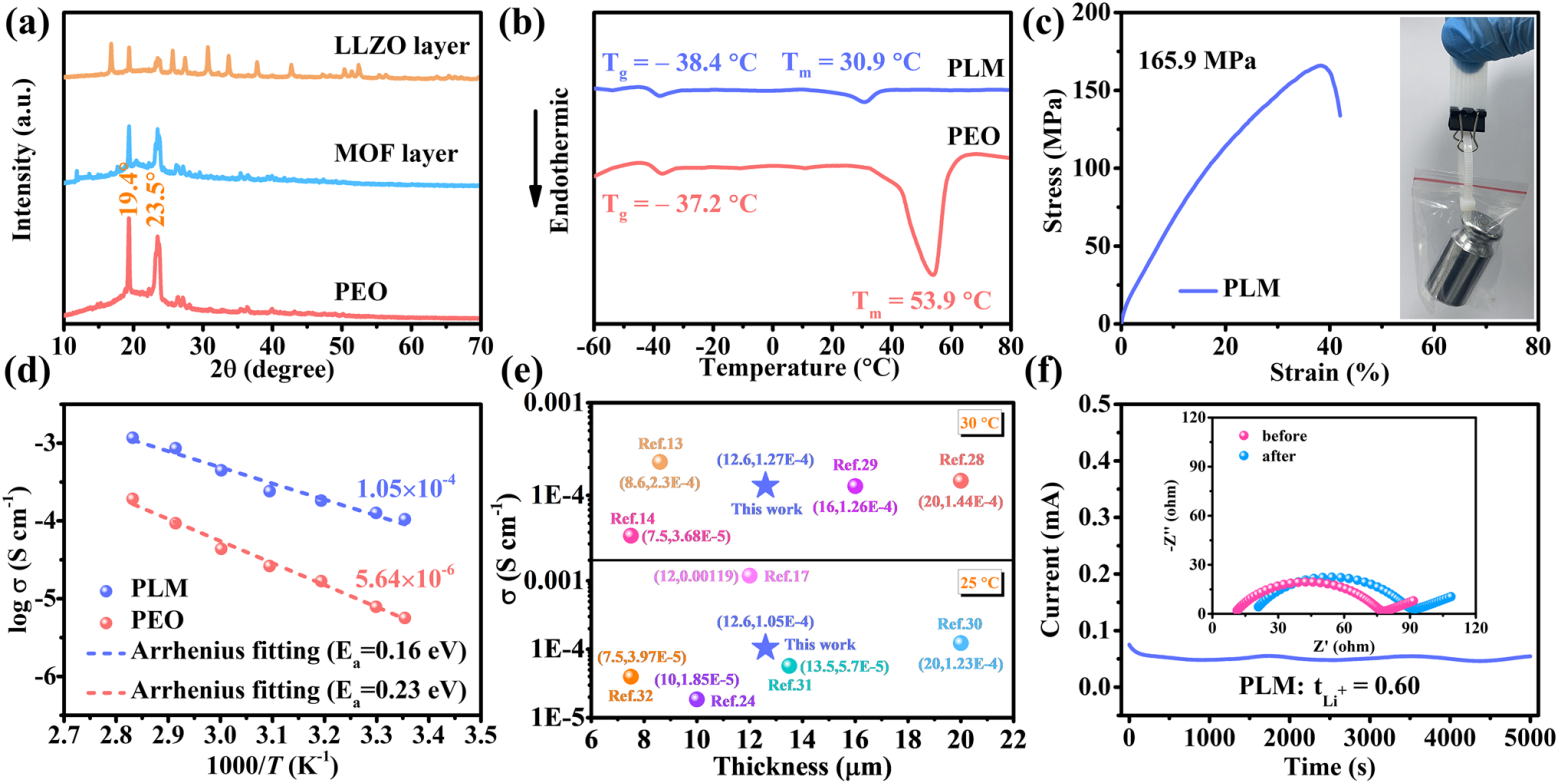
**a** XRD patterns of PEO, MOF layer and LLZO layer. **b** DSC curves of PEO and PLM. **c** Stress–strain curve of PLM. The inset of **c** shows that PLM can pull up a 100 g weight. **d** Ion conductivity of PEO and PLM. **e** Thickness and ionic conductivity (25 and 30 °C) of ultrathin PLM electrolytes compared with recently reported electrolytes. **f** Li^+^ transference number of PLM

The phase composition of the electrolyte was investigated by XRD (Fig. 2a) and the sharp diffraction peaks at 19.4° and 23.5° were attributed to the characteristic peaks of PEO electrolyte [21]. The intensity of the diffraction peaks of PEO are significantly reduced for both MOF and LLZO layers compared with PEO electrolyte, indicating that the introduction of functional fillers significantly reduces the crystallinity of the PEO matrix [22]. The increase of the amorphous region is crucial for the enhancement of the ionic conductivity of CSEs, which can be further confirmed by the differential scanning calorimeter (DSC) [23]. From Fig. 2b, the glass transition temperature (T_g_) and melting temperature (T_m_) of the asymmetric PLM electrolyte are significantly lower than those of the PEO electrolyte. In addition, the characteristic peaks of MOF and LLZO can be detected simultaneously in the XRD pattern, indicating a well-preserved filler structure. Electrolytes are required to have sufficient mechanical strength for practical applications to satisfy structural integrity and prevent Li dendrite punctures [24]. The mechanical property of the electrolyte was analyzed by tensile testing (Fig. 2c). With the introduction of PE support, the mechanical properties of the PLM electrolyte were significantly improved. The tensile strength of PLM electrolyte is ≈165.9 MPa, which is much higher than that of the PEO electrolyte (Fig. S10, tensile strength of 0.48 MPa). For the visual demonstration (inset), the PLM electrolyte was able to pull up a 100 g weight easily. In addition, the thermal stability of the PLM was also investigated. As shown in Fig. S11, when the temperature was increased to 130 °C, the PE separator underwent severe shrinkage and the PEO electrolyte softened and became transparent. In contrast, PLM was able to maintain the dimensional stability better. The improved dimensional stability ensures the safety of SSLMBs for high-temperature operation.

Figures 2d and S12 show the variation of electrolyte ionic conductivity with temperature. Due to the high crystallinity of the polymer matrix at room temperature, PEO electrolytes exhibit low ionic conductivity (5.64 × 10^–6^ S cm^−1^). In the LLZO and MOF layers, the amorphous region is increased to facilitate ionic conduction due to the reduced crystallinity of the polymer matrix. In addition, the conductive interface between the filler and the polymer matrix creates more ionic conduction paths to accelerate ionic transport, and these factors endow the PLM electrolyte a higher ionic conductivity of 1.05 × 10^–4^ S cm^−1^ [25, 26]. According to the Arrhenius relation, PLM shows lower activation energy (*E*_a_) than PEO, which suggests a lower migration barrier and fast ion migration kinetics of Li^+^ in PLM [7, 27]. It is noteworthy that ultrathin electrolytes contribute to lower ohmic resistance and improved battery performance, as electrolytes with the smaller thicknesses have higher area-normalized ionic conductance (*G* = *σA/l*, where *G*, *σ*, *A* and *l* denote ionic conductance, ionic conductivity, area and thickness of the electrolyte, respectively) and the shorter time required for ion transport in ultrathin electrolytes (*t* = *l*^*2*^/*D*, where *t*, *l* and *D* denote Li diffusion time, thickness of the electrolyte and Li diffusion constant, respectively) [11]. Figure 2e compares the relationship between thickness and ionic conductivity of recently reported ultrathin electrolytes, and it can be found that our designed PLM electrolyte outperforms most electrolyte systems with similar thickness [13, 14, 17, 24, 28–32]. Moreover, due to the anion-immobilization by LLZO and MOFs nanoparticles [33, 34], the Li^+^ transference number of PLM electrolyte (0.60) is significantly higher (Fig. 2f) than that of PEO electrolyte (0.16, Fig. S13). The high Li^+^ transference number will help alleviate the interfacial polarization and promote the fast charging capability of the SSLMBs [34].

### Interface Analysis of Li Symmetric Batteries

**Fig. 3:**
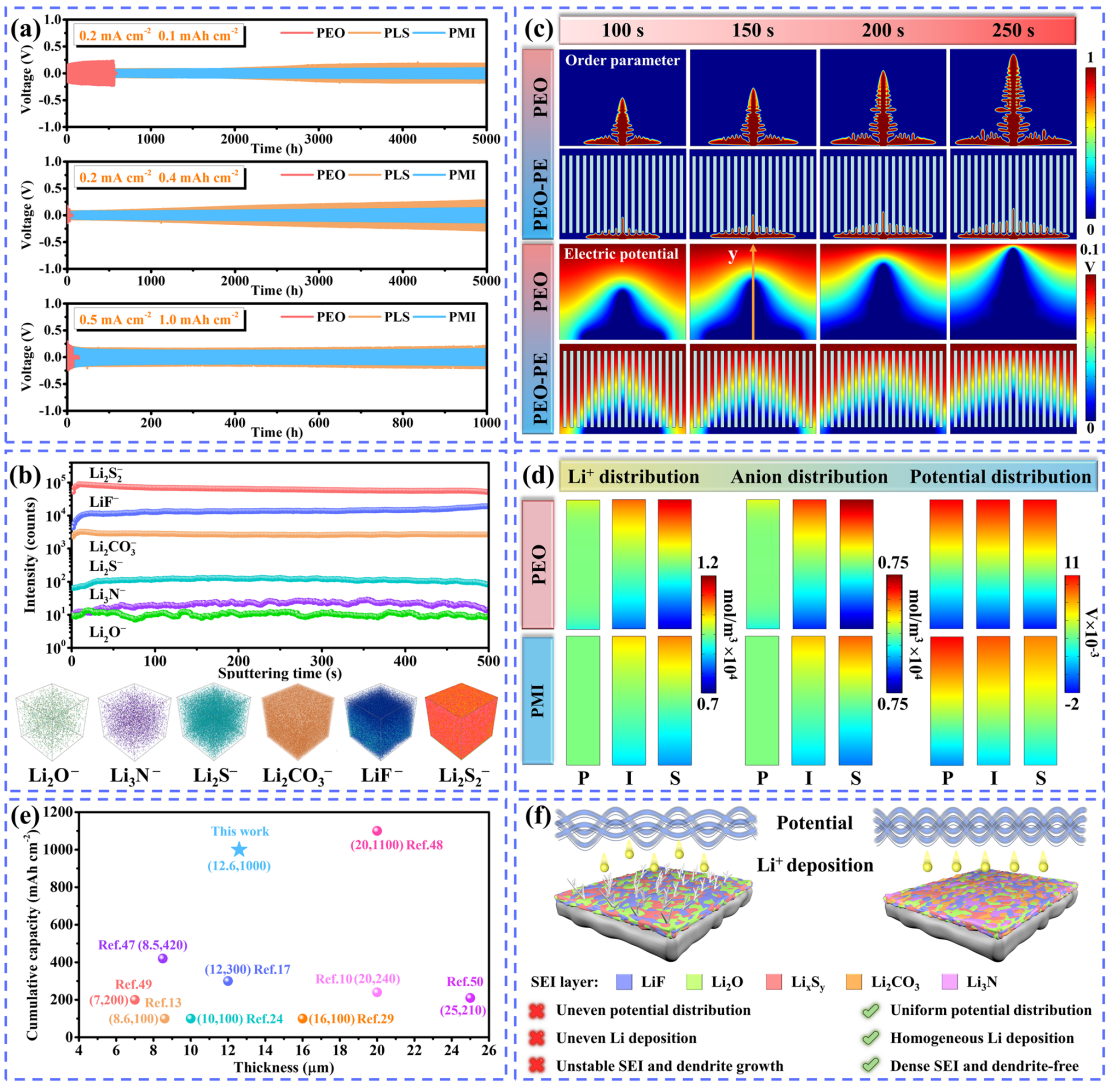
**a** Li plating/stripping curves of PEO, PLS and PMI. **b** TOF–SIMS depth profiles for the PMI/Li interface and the corresponding 3D view. **c** Snapshots of the PEO and PEO-PE electrolyte order parameters and potential distributions as a function of time. **d** Distribution of Li^+^, anion and potential in PEO and PMI (P: pristine state, I: intermediate state, S: steady state). **e** Comparison of electrolyte thickness and cumulative capacity with previous reported. **f** Schematic diagram of the stability to Li metal (The left side represents the Li/PEO interface and the right side represents the Li/PMI interface)

To demonstrate the difference in the stability of different electrolytes for Li anode, MOF (PMI), LLZO (PLS), PEO-PE and PEO/SN-PE symmetric electrolytes were prepared in the same way, and the ionic conductivities of the different electrolytes are shown in Fig. S14. Li symmetric batteries were assembled to verify the stability of the electrolyte for Li anode. The polarization potential of the PEO electrolyte increases with time. Due to insufficient mechanical strength of the PEO matrix, Li/PEO/Li batteries experience a short circuit (Fig. 3a) after cycling for 566 h. When PE separator was introduced in the PEO matrix (PEO-PE), the cycle life of Li symmetric batteries was extended to 1400 h (Fig. S15), which confirmed the necessity of introducing PE separator in the polymer matrix for inhibiting Li dendrite growth. Further increasing the deposition capacity and current density, the Li/PEO/Li batteries produced a high overpotential and the batteries short-circuited quickly (Fig. S17), indicating that a large space charge layer was generated at the PEO/Li interface under the high deposition capacity [36]. Although the Li symmetric batteries with PLS electrolyte operated without short circuit, the PMI electrolyte showed a smaller polarization potential (Fig. S17) and a more stable cycling profile (Fig. S18), which may be related to the incompatibility between SN and Li metal in the PLS electrolyte. The SN continuously reacts with the Li metal during long-term cycling, leading to severe damage at the interface [37]. The plating/stripping curves of Li symmetric batteries revealed that the PMI electrolyte has excellent stability to Li metal, exhibiting an ultralong cycle life (5000 h) and a low overpotential, and can still cycle stably for 1000 h even at 0.5 mA cm^−2^/1.0 mAh cm^−2^, which is significantly better than that of Li/PEO-PE/Li and Li/PEO/SN-PE/Li batteries (Fig. S19). This may be that MOF with porous structures can act as ion sieves that preferentially promote cation transfer, thus prevent the growth of Li dendrites [38].

The morphology of the Li anode was observed by disassembling the Li symmetric batteries after cycling to visually reflect the effect of the electrolyte on the inhibition of Li dendrites. The results showed that the Li anode surface remained smooth and flat without dendrite formation in Li/PMI/Li batteries under different test conditions (Figs. S20–S22). This further implies that the MOF layer can play an important role in promoting the uniform deposition of Li and inhibiting the growth of Li dendrites [39].

X-ray photoelectron spectroscopy (XPS) and time of flight secondary ion mass spectrometry (TOF–SIMS) measurements were performed on the cycled (400 h) electrolyte to investigate the SEI composition. Figure S23 compare the XPS spectra of PMI and PEO electrolytes after cycling. More C–C/C–H bonds are observed in the C 1*s* spectra of the PEO electrolyte, implying a poorer electrochemical stability of the PEO electrolyte [40]. Abundant LiF, Li_3_N, Li_2_CO_3_, Li_2_S_2_/Li_2_S/Li_2_S_x_, and Li_2_O were observed in the PMI electrolyte, which suggests the formation of a Li-rich SEI layer between the Li anode and the MOF layer. Specifically, LiF is considered as a key component in SEI and plays an important role in controlling the uniform Li^+^ transport and deposition [40]. Li_3_N has a very low Li^+^ diffusion barrier, which ensures rapid Li^+^ crossing of the Li anode/electrolyte interface [41]. The synergistic effect of Li_2_CO_3_ and LiF can improve the Li^+^ conductivity in the system [42]. Inorganic sulfides contribute to an interface with low interfacial resistance and improve SEI stability [27]. Li_2_O can reduce the Li nucleation overpotential and extend the lifetime of the battery [43]. The TOF–SIMS depth profiling further detects the interfacial phase composition (Fig. 3b). As the 3D view shows, a SEI layer with a certain thickness is formed at the interface. The stabilized SEI layer not only ensures a good electric field distribution, promotes uniform Li deposition and inhibits Li dendrite growth, but also stabilizes the Li anode/electrolyte interface, resulting in a flat morphology of the cycled Li anode. Compared with PMI electrolytes, PEO electrolytes have a lower content of Li-rich compounds, which is not sufficient to form a strong and stable SEI, resulting in poor stability of the Li anode and causes excessive Li dendrite growth thus short-circuiting the battery.

To further clarify the excellent interfacial stability of the PMI electrolyte to the Li anode, simulation calculations were performed by applying COMSOL Multiphysics (Figs. S24–S26, Table S1). Figure 3c shows the snapshots of the PEO and PEO-PE electrolyte order parameters and potential distributions as a function of time. During the electrodeposition process, Li dendrites grow abnormally large in the PEO electrolyte. Due to the improved mechanical properties of the PEO-PE electrolyte, the dendrite growth becomes significantly slower, and the Li dendrite volume tends to be smaller. In addition, a large potential gradient is also found at the tip of the dendrite in the potential distribution map. The potential distribution at different positions is plotted from the central axis of the electric potential distribution map (Fig. S27). The potential changes faster in the PEO electrolyte, which also forces a larger overpotential to make Li dendrites grow wantonly [44, 45]. Figure 3d further analyzes the evolution of Li^+^ concentration and potential in different states. Driven by an electric field, positive and negative ions pass through the electrolyte and aggregate at both ends. Since there is no effective mediator in the PEO electrolyte to relieve this imbalance, larger Li and anion concentrations are observed, resulting in a larger potential. For the PMI electrolyte, the PE framework and the MOF layer jointly ensure that the Li/anion concentration and potential gradient inside the electrolyte are smaller, thereby inducing dendrite-free Li deposition [46]. In summary, the theoretical simulations are in high agreement with the experimental results, confirming the superiority of the MOF layer in regulating the uniform Li deposition. Usually, the ability of electrolytes to inhibit Li dendrite growth is also affected by their thickness. The cumulative cycling capacity is calculated by multiplying the current density and cycling time of the Li symmetric batteries to evaluate the inhibitory ability of the electrolyte on the Li dendrites [17]. As shown in Fig. 3e, our as-prepared electrolytes show significant competitiveness beyond most of the reports [10, 13, 17, 24, 29, 47–50]. Aided by the evenly distributed potential and the dense SEI composed of organic–inorganic components (Fig. 3f), a stable electrochemical process between the electrolyte and Li anode is realized, thus allowing Li symmetric batteries with ultralong cycle life.

### Electrochemical Performances of SSLMBs

**Fig. 4:**
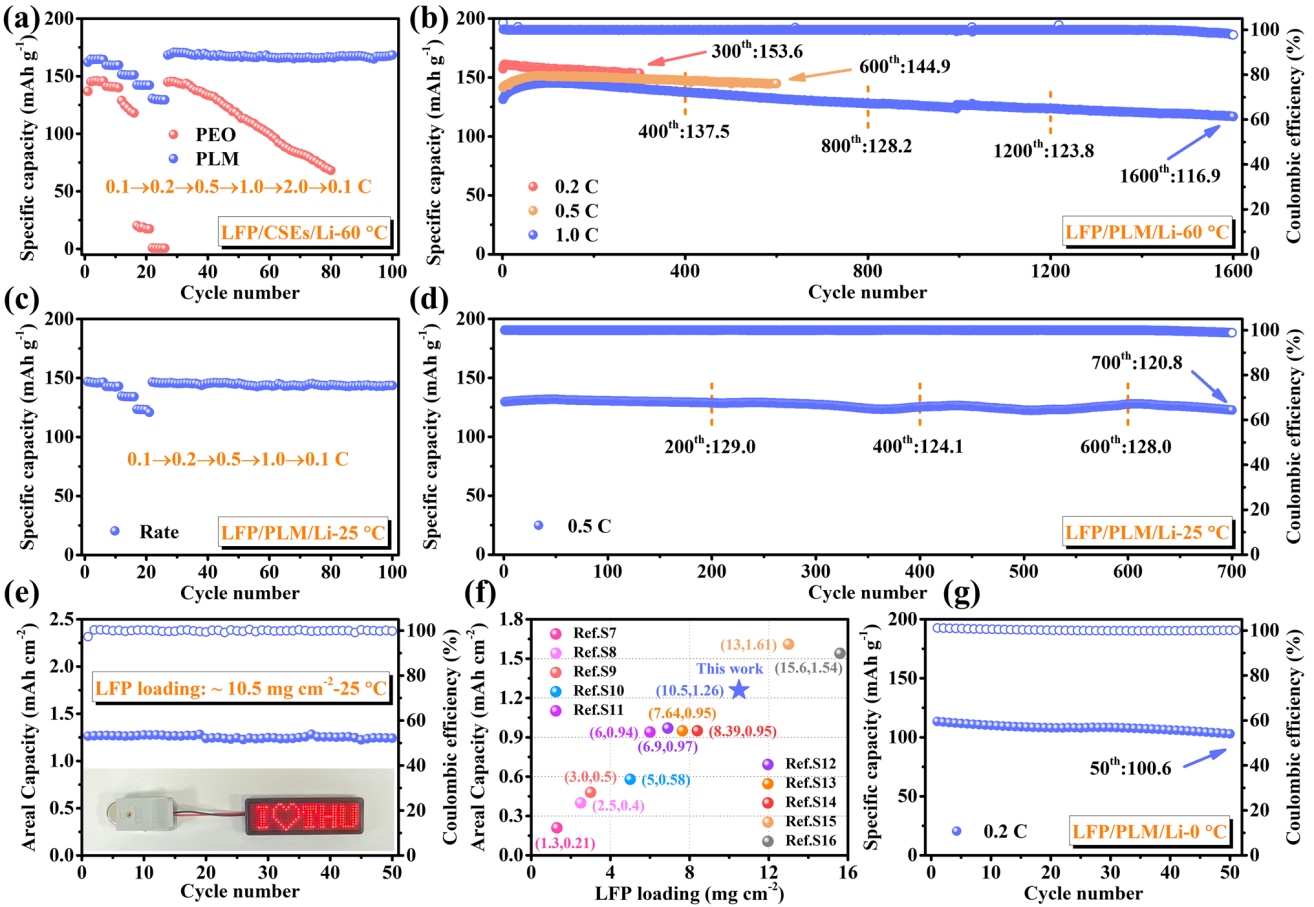
Electrochemical performances of LFP-based SSLMBs at 60 °C. **a** Rate performance. **b** Cycling performance at 0.2, 0.5 and 1.0 C. Electrochemical performances of LFP-based SSLMBs at 25 °C. **c** Rate performance. **d** Cycling performance at 0.5 C. **e** Cycling performance at 0.1 C with LFP loading ~ 10.5 mg cm^−2^. **f** Relationship between LFP loading and areal capacity, and compared with previous studies. **g** Cycling performance at 0 °C (0.2 C)

To demonstrate the potential applications of PLM electrolytes for SSLMB, the electrochemical performance of LFP batteries was first tested at high temperature (60 °C). LFP/PLM/Li battery exhibited superior rate performance (Fig. 4a), providing a specific capacity of 129.8 mAh g^−1^ even at 2.0 C. When the rate returns to 0.1 C, the specific capacity can be fully recovered (167.3 mAh g^−1^). In contrast, LFP/PEO/Li battery cannot provide sufficient specific capacity at high rates. Since the PLM electrolyte has higher ionic conductivity and Li^+^ transference number, LFP/PLM/Li exhibits lower polarization potentials in different rate tests [51] (Fig. S28). In addition, the ultrathin characteristics and the low interfacial impedance (Fig. S29) of the PLM electrolyte ensures fast Li^+^ transport at high rate of SSLMBs [15]. A further comparison of the long-term cyclic stability of the two SSLMBs is performed. The specific capacity of LFP/PEO/Li battery gradually decreases with the extension of the cycle number (Fig. S30), exhibiting poor cycling stability, excessive polarization potential (Fig. S31) and interfacial impedance (Fig. S32). This instability is due to poor interfacial contact and Li dendrite growth in PEO electrolyte, leading to capacity fading [16, 52]. In contrast, with the help of the MOF/LLZO layer and PE separator, the PLM electrolyte achieves low interfacial impedance and dendrite suppression, allowing the stable operation of SSLMBs at different rate (0.2 C/153.6 mAh g^−1^-300th, 0.5 C/144.9 mAh g^−1^-600th). Even after 1600 cycles at 1.0 C (Fig. 4b), it can still provide a specific capacity of 116.9 mAh g^−1^ with an average specific capacity decay as low as 1.78% (per cycle), which is better than the recently reported SSLMBs [14–16, 24, 36, 47, 53–55] (Fig. S33). In addition, the design of the asymmetric PLM electrolyte significantly improved the cycling performance of the PE separator reinforced polymer matrix electrolyte at 1.0 C (Fig. S34). The potential distributions of SSLMBs configured with different electrolytes (PEO and PLM) were simulated by COMSOL Multiphysics (Fig. S35) to further reveal the essence of the excellent electrochemical performance of asymmetric electrolytes. Larger potential gradient changes are observed in the 2D cross-sectional view of SSLMBs with PEO electrolytes, especially higher potentials exist at the LFP/PEO interface. These factors lead to the formation of a space charge layer, resulting in severe polarization of the battery [10]. Benefiting from the designed PLM electrolyte with asymmetric structure, a more uniform potential is generated, which will alleviate the ion concentration polarization and enhance the cycle stability.

Subsequently, the electrochemical performance of the LFP/PLM/Li batteries at room temperature (RT, 25 °C) was tested to highlight the superiority of the PLM electrolyte. As shown in Fig. 4c, the average discharge specific capacities of the batteries at 0.1, 0.2, 0.5, and 1.0 C are 146.3, 142.8, 134.3, and 122.8 mAh g^−1^, respectively. When the rate returns to 0.1 C, the average discharge capacity reaches 144.3 mAh g^−1^. The long-term cycling stability of SSLMBs was further explored, providing reversible capacities of 135.1 and 120.8 mAh g^−1^ after 200 cycles (0.2 C, Fig. S36) and 700 cycles (0.5 C, Fig. 4d), respectively. These results demonstrate the excellent rate performance and long-term cycling stability of the LFP/PLM/Li batteries even at room temperature, which inspired us to explore the high-load LFP batteries. When the LFP loading was increased to 10.5 mg cm^−2^, the SSLMBs deliver an areal capacity of 1.26 mAh cm^−2^ at 0.1 C (Fig. 4e), with almost no capacity fading after 50 cycles. In addition, the SSLMBs exhibited a clear charge/discharge plateau throughout the cycle (Fig. S37). Figure 4f plots the relationship between LFP loading and areal capacity, and compared with previous studies (Table S2), our designed PLM electrolyte is more competitive in terms of areal capacity, thickness and operating temperature. Excitingly, even at low temperatures (0 °C) SSLMBs can still operate properly. Despite the increased overpotential compared with room temperature and high temperature (Fig. S38), the charge–discharge curve of the LFP/PLM/Li battery is smooth and can still provide a specific capacity of 100.6 mAh g^−1^ after 50 cycles (Fig. 4g). This demonstrates the promise of PLM electrolytes for potential future applications in low-temperature operating conditions, which can be attributed to the following factors: (1) the ultra-thin PLM electrolyte design shortens the Li^+^ transport path; (2) SN and ionic liquids in the LLZO and MOF layers help to facilitate rapid ion transport [56, 57]; (3) Trace amount of liquid electrolyte enhances the wettability between electrolyte and anode and improves ion transport inside the electrode [58].

**Fig. 5:**
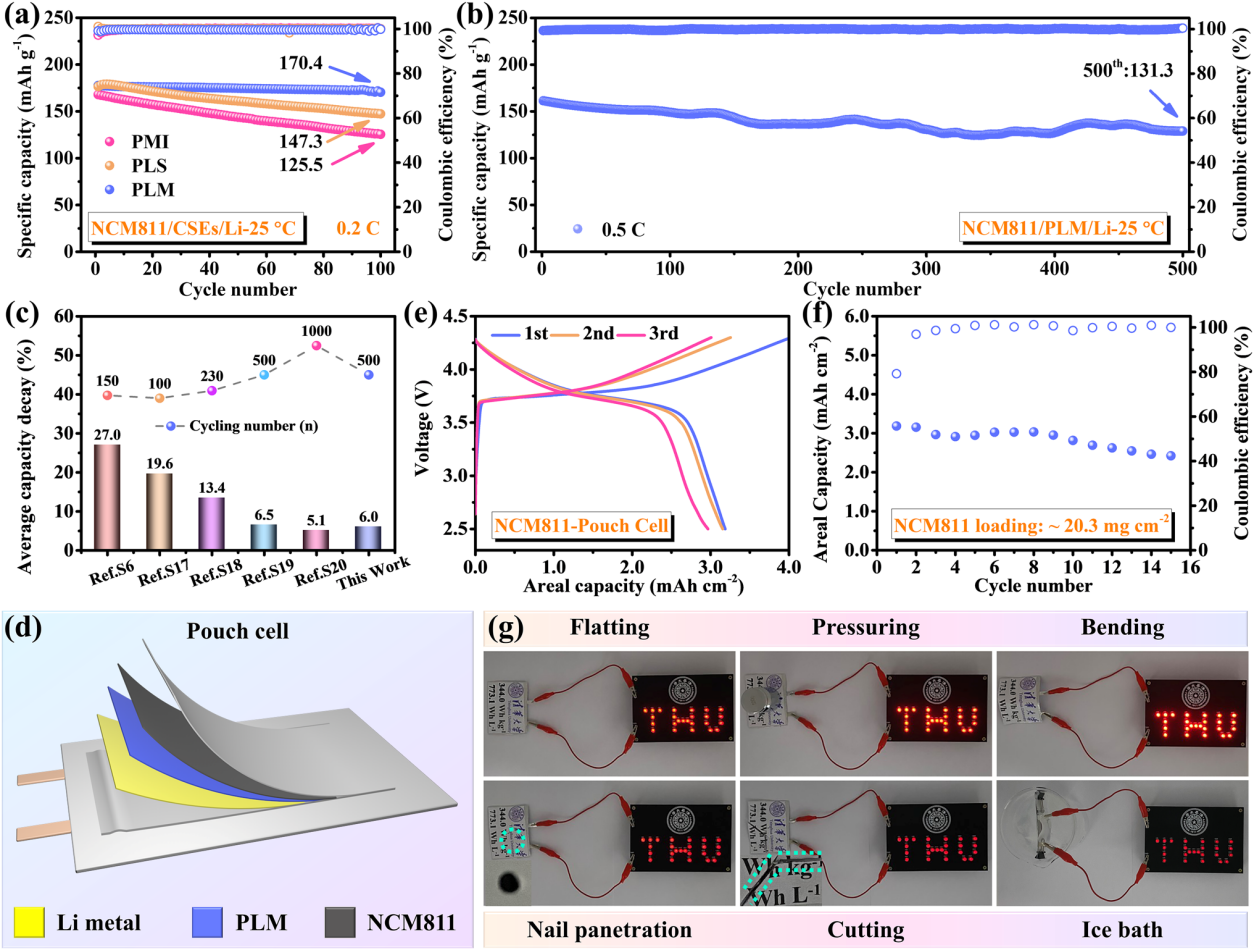
Electrochemical performances of NCM811-based SSLMBs at 25 °C. **a** Cycling performance of NCM811/CSEs/Li at 0.2 C. **b** Cycling performance of NCM811/PLM/Li at 0.2 C. **c** The comparison of NCM811/PLM/Li cycling performance with previous reports. **d** Schematic diagram of pouch cell. **e** Galvanostatic charge/discharg curves of NCM811/PLM/Li pouch cell. **f** Cycling performance of NCM811/CSEs/Li pouch cells. **g** The pouch cell light up the LED sign in different states (Flatting, pressuring, bending, nail penetration, cutting and ice bath)

The original intention of the asymmetric structure design was not only to form a good interface with the Li anode, but more importantly to be able to meet the matching of the electrolyte with the high-voltage cathode to improve the energy density of the SSLMBs. Benefiting from the excellent electrochemical stability of LLZO [53], the electrochemical stabilization window (Fig. S39) of the electrolyte is significantly broadened, in which the electrochemical stability window of PLS reaches 5.22 V, higher than that of PLM (5.11 V) and PMI (4.78 V). The dual-interface design of the PLM electrolyte fully exploits its interfacial compatibility with Li anode and high-voltage cathode, and the embedded PE separator is expected to suppress Li dendrite growth, these factors are considered to be the key to ensure the stable operation of high-voltage SSLMBs. To further illustrate the necessity of asymmetric structural design, Fig. 5a compares the cycling stability of NCM811 based SSLMBs assembled with PMI, PLS, and PLM electrolytes. In particular, the NCM811/PLM/Li battery delivered a specific capacity of 170.4 mAh g^−1^ after 100 cycles with 95.9% capacity retention, which is significantly better than that of NCM811/PMI/Li (125.5 mAh g^−1^, 74.7%) and NCM811/PLS/Li (147.3 mAh g^−1^, 83.5%). By disassembling the cycled SSLMBs after 50 cycles at 0.2 C, the surface morphologies of the NCM cathode and Li anode were observed (Fig. S40) to reflect the existing interfacial issues. It can be observed that the PLS electrolyte is unstable towards the anode interface, and the Li metal surface is slightly corroded by the presence of SN. While the PMI electrolyte is unstable to the cathode interface and electrolyte decomposition occurs at the NCM cathode surface. In sharp contrast, and achieved a satisfactory cathode/anode interface. In addition, the NCM811/PLM/Li battery exhibited minimal overpotential (Fig. S41) and interface impedance (Fig. S42) after 50 cycles. The composition of the cathode electrolyte interface (CEI) was explored by comparing the XPS spectra of the NCM anode before and after cycling. As shown in Fig. S43, a CEI layer enriched with LiF and Li_3_N was formed at the anode/electrolyte interface, where -C≡N corresponds to SN, which proves that salt anions and SN participate in the interfacial reaction. Notably, the La atoms in LLZO can complex with the N atoms in SN to prevent free SN molecules from diffusing to the Li anode side to corrode the Li surface [59]. The formation of the CEI layer contributes to the stable high-voltage cycling performance [37]. The long-term cycling stability of the NCM811/PLM/Li battery at 0.5 C was further evaluated. As shown in Fig. 5b, a specific capacity of 131.3 mAh g^−1^ maintained after 500 cycles. This well-designed asymmetric PLM electrolyte enables the cycle stability and cycle life of high-voltage SSLMBs to be at the forefront of recent reports (Fig. 5c, Table S3).

Actual working performance was tested by assembling pouch cells with PLM electrolyte, NCM811 cathode (20.3 mg cm^−2^) and Li belt (40 μm). Figure 5d shows that the pouch cell has a stable charge–discharge curve and can provide an areal capacity of 3.19 mAh cm^−2^ (Fig. 5e), with a corresponding gravimetric/volume energy density of 344.0 Wh kg^−1^/773.1 Wh L^−1^ (Table S4), which is important for advancing the practicality of SSLMBs. The pouch cells can light up the LED sign even under vicious conditions (Fig. 5f), demonstrating safety and reliability.

## Conclusions

In summary, the rational design of ultrathin asymmetric PLM electrolytes achieves the excellent interfacial properties of SSLMBs. The PE separator framework significantly improves the mechanical strength of the polymer matrix. The LLZO and MOF layers attached to both sides of the PE separator not only improve the ionic conductivity of the polymer matrix, but also stabilize the anode and cathode interfaces. Specifically, the MOF layer promotes the stable SEI layer formation, homogenizes the Li flux to achieve a more stable potential. The LLZO layer provides high-voltage stability and forms an ultra-durable CEI layer, enabling stable cycling performance of PEO-based electrolytes with high-voltage cathodes. In addition, the well-designed PLM electrolyte enables SSLMBs to achieve excellent charge/discharge performance over a wide temperature range. More importantly, the equipped pouch cells can work even under extreme conditions, demonstrating practical application value. The design concept of the ultrathin asymmetric electrolyte proposed in this study, with its tractable operation and large-scale practicality, which provides guidance for promoting the development of high-energy–density and high-safety SSLMBs.

## Supplementary Information

Below is the link to the electronic supplementary material.

Supplementary file1 (PDF 4216 KB)
