# Supplementary material for: 12.6 μm-Thick Asymmetric Composite Electrolyte with Superior Interfacial Stability for Solid-State Lithium-Metal Batteries
Source: Nanomicro Lett. 2024 Apr 26;16:181. doi: 10.1007/s40820-024-01389-2 (PMC11052750; doi:10.1007/s40820-024-01389-2)
Supplement: Supplementary file 1 — Supplementary file1 (PDF 4216 KB) [file 40820_2024_1389_MOESM1_ESM.pdf]

Supporting Information for

12.6 µm-Thick Asymmetric Composite Electrolyte with Superior Interfacial Stability for Solid-State Lithium-Metal Batteries

Zheng Zhang[1], Jingren Gou[1], Kaixuan Cui[1], Xin Zhang[1], Yujian Yao[1], Suqing Wang[2,*], Haihui Wang[1,*]

[1] Beijing Key Laboratory for Membrane Materials and Engineering, Department of Chemical Engineering, Tsinghua University, Beijing 100084, P. R. China

[2] School of Chemistry and Chemical Engineering, South China University of Technology, Guangzhou 510000, P. R. China

*Corresponding authors. E-mail: cehhwang@tsinghua.edu.cn (Haihui Wang); cesqwang@scut.edu.cn (Suqing Wang)

S1 Experimental

S1.1 Preparation of LLZO Ceramics

Materials Li_2CO_3 (99.9%, Aladdin), La_2O_3 (99.9%, Aladdin), ZrO_2 (99.9%, Aladdin), $Cu(NO_3)_2·3H_2O$ (99%, Aladdin), polyvinylpyrrolidone (PVP−K30, Sigma-Aldrich), 1, 3, 5−benzenetri−carboxylic acid (H_3BTC, 98%, Aladdin), 1-Ethyl-3-methylimidazolium bis(trifluoromethylsulfonyl)imide (EMIMTFSI, 97%, Aladdin), PEO (M_w=600,000, Aladdin), succinonitrile (SN, 99%, Aladdin), Bis(trifluoromethane)sulfonimide lithium salt (LiTFSI, 99%, Aladdin), methanol (AR, 99.5%, Aladdin), acetonitrile (AR, 99%, Aladdin), N-methyl-2-pyrrolidone (NMP, 99.5%, Aladdin).

Preparation of LLZO Ceramics $Li_7La_3Zr_2O_{12}$ (LLZO) ceramics are prepared by solid-phase sintering. Typically, La_2O_3 is pre-fired at 900 °C for 12 h to remove traces of moisture and adsorbed CO_2 and to prevent the formation of $La_2Zr_2O_7$ impurities. The stoichiometric Li_2CO_3 (42 mmol), ZrO_2 (28 mmol) and pre-fired precursor (21 mmol) La_2O_3 were used as a starting material, and an excess of 10 wt% Li_2CO_3 was added to compensate for the loss of Li during the sintering process. The raw material was ground at 500 rpm for 12 h under isopropanol. After ball milling, precipitation was collected by centrifugation and dried in a 60 °C vacuum oven for further use. The dried mixture was sintered at 900 °C for 12 h.

Preparation of MOFs $Cu(NO_3)_2·3H_2O$ (9.0 g) and PVP (4.0 g) were dissolved in 500 mL of anhydrous methanol to obtain solution A. H_3BTC (4.3 g) in 500 mL of anhydrous methanol to obtain B solution. Then, solution B was added to solution A under continuous stirring, and stirring was continued for 10 min to mix the solution uniformly, it was left at room temperature for 24 h. Finally, the precipitate was collected by centrifugation and dried in a vacuum oven at 120 °C to obtain MOFs. The ion-conducting Li-IL@MOFs were obtained by grinding 0.10 g of MOFs filled with 110 µL of Li-IL (LiTFSI/EMIMTFSI, 2 M) in a glove box, and the mixture was placed in a vacuum oven at 120 °C (12 h) for thermal diffusion. The as-prepared Li-IL@MOFs were stored in a glove box to avoid the interference of oxygen and moisture for subsequent preparation of the MOF layers.

S1.2 Structure Characterizations

X−ray diffraction (XRD) data were recorded by Rigaku SmartLab. Morphology and structure were measured by scanning electron microscope (SEM, Zeiss Merlin Compact) and transmission electron microscopy (TEM, Talos F200X G2). X-ray photoelectron spectroscopy (XPS) was characterized on Thermo Scientific K-Alpha (Al-Kα). Thermogravimetric analysis (TGA) was carried out in air atmosphere with a heating rate of 10 °C min^{-1} on a Mettler Toledo TGA STAR system. Differential scanning calorimetry (DSC, Mettler−Toledo) tests were performed in N_2 at a heating rate of 5 °C min^{-1}. Time of flight secondary ion mass spectrometry (TOF-SIMS) was employed to detect the 3D components distribution of interface by ION-TOF GmbH TOF-SIMS5-100. The stress-strain curves were measured by Instron 5969 with sample dimension of 10 cm × 2 cm × 12.6 μm at a constant speed of 1 mm min^{-1}.

S1.3 Electrochemical Characterization

Ionic conductivity (IC), linear sweep voltammetry (LSV) and Li^+ transference number (t_{Li^+},) were collected with a Gamry Interface 1010 electrochemical workstation. For the IC, the CSEs was sandwiched between two stainless steel plates at temperatures from 25 to 80 °C and the frequency range was 0.1 to 10^5 Hz (SS/CSEs/SS). The IC was calculated by the following formula.

$$\sigma = \frac{l}{RA} \quad \text{(S1)}$$

where l represents the thickness of the CSEs, R represents the impedance, and A is the area of the stainless−steel.

$$\sigma_T = Aexp(\frac{-E_a}{RT}) \quad \text{(S2)}$$

where σ_T is the ionic conductivity at different temperatures, E_a is the activation energy, T is the absolute temperature, and A is the pre−exponential factor. The LSV test was performed by assembling a Li/CSEs/SS cell at a scan rate of 0.5 mV s^{-1} from open circuit voltage to 6.0 V to evaluate the electrochemical stability of the CSE to lithium metal. t_{Li^+} was measured by the chronoamperometry and calculated according to following formula.

$$t_{Li^+} = \frac{I_{SS}(\Delta V - I_0 R_0)}{I_0(\Delta V - I_{SS} R_{SS})} \quad \text{(S3)}$$

Where I_0 is the initial current, I_{SS} is the steady−state current, R_0 is the interface impedance before polarization, and R_{SS} is the interface impedance after polarization, ΔV is voltage bias (10 mV).

Solid−state lithium metal battery (SSLMB) is prepared by using $LiFePO_4$ (LFP), $LiNi_{0.8}Mn_{0.1}Co_{0.1}O_2$ (NCM811), CSEs, Li metal. The cathode contained 80 wt% active materials, 10 wt% super P, and 10 wt% PVDF. Unless otherwise specified, the active material weight of cathode was about 2.0 mg cm^{-2}. To enable the SSLMBs to charge and discharge at room temperature and low

temperature, 2 μL cm^{-2} liquid electrolyte (1.0 mol L^{-1} $LiPF_6$/ethylene carbonate(EC):diethylcarbonate(DEC): ethylmethylcarbonat(EMC) = 1:1:1, by volume) was used to infiltrate the cathode side to enhance interfacial contact. 20.3 mg cm^{-2} of NCM811 cathode was used to assemble the pouch cell, and the cathode was infiltrated with 5 μL cm^{-2} liquid electrolyte. The $LiFePO_4$/CSEs/Li SSLMBs were charged and discharged between 2.7–3.8 V. The NCM811/CSEs/Li SSLMBs were charged and discharged between 2.5–4.3 V. The long-term cycling tests of Li symmetric batteries were performed by galvanostatic charging and discharging at current densities/time of 0.2 mA cm^{-2}/0.5 h, 0.2 mA cm^{-2}/2.0 h, and 0.5 mA cm^{-2}/2.0 h, respectively.

S2 Supplementary Figures

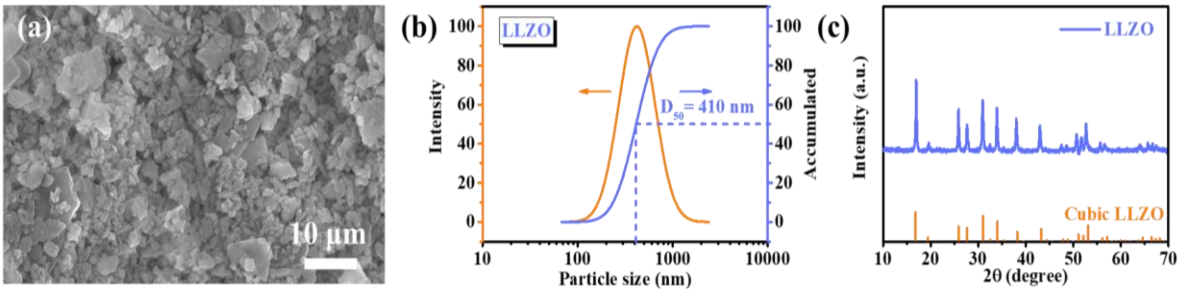

Fig. S1 (**a**) SEM images of LLZO, (**b**) particle size distribution curve of LLZO, (**c**) XRD pattern of LLZO

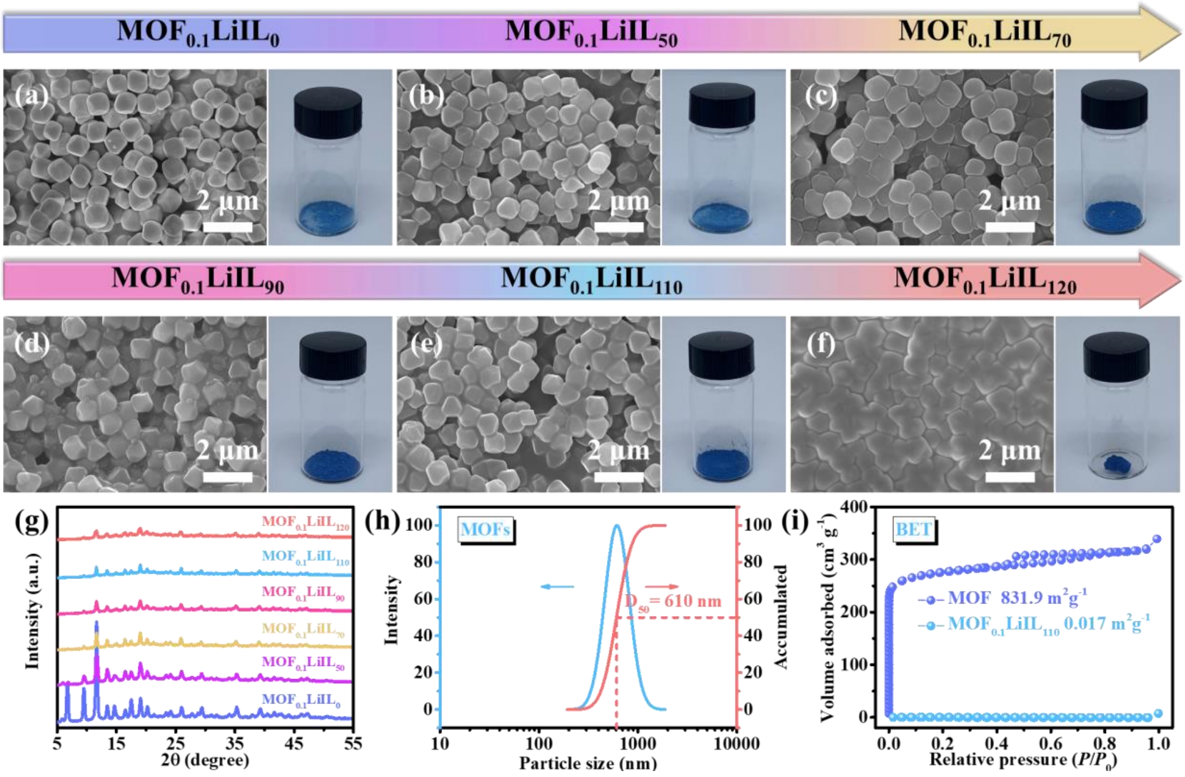

Fig. S2 (**a-f**) SEM images and optical photographs of $MOF_{0.1}LiIL_x$ composites, (**g**) XRD pattern of $MOF_{0.1}LiIL_x$ composites, (**h**) particle size distribution curve of MOFs, (**i**) BET curves of $MOF_{0.1}LiIL_0$ and $MOF_{0.1}LiIL_{110}$

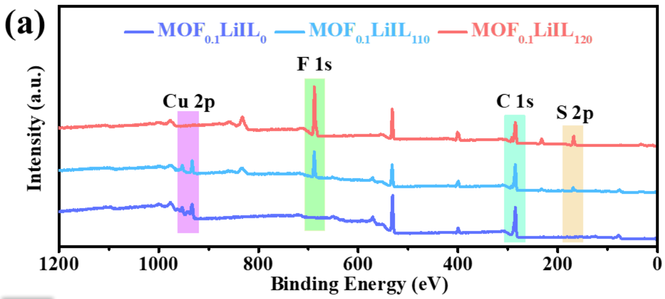

Element Atomic(%)	$MOF_{0.1}$ $LiIL_0$	$MOF_{0.1}$ $LiIL_{110}$	$MOF_{0.1}$ $LiIL_{120}$
S	0.13	5.48	9.64
F	0.16	18.74	33.24
Cu	7.82	3.60	0.19
C	91.89	72.18	56.93

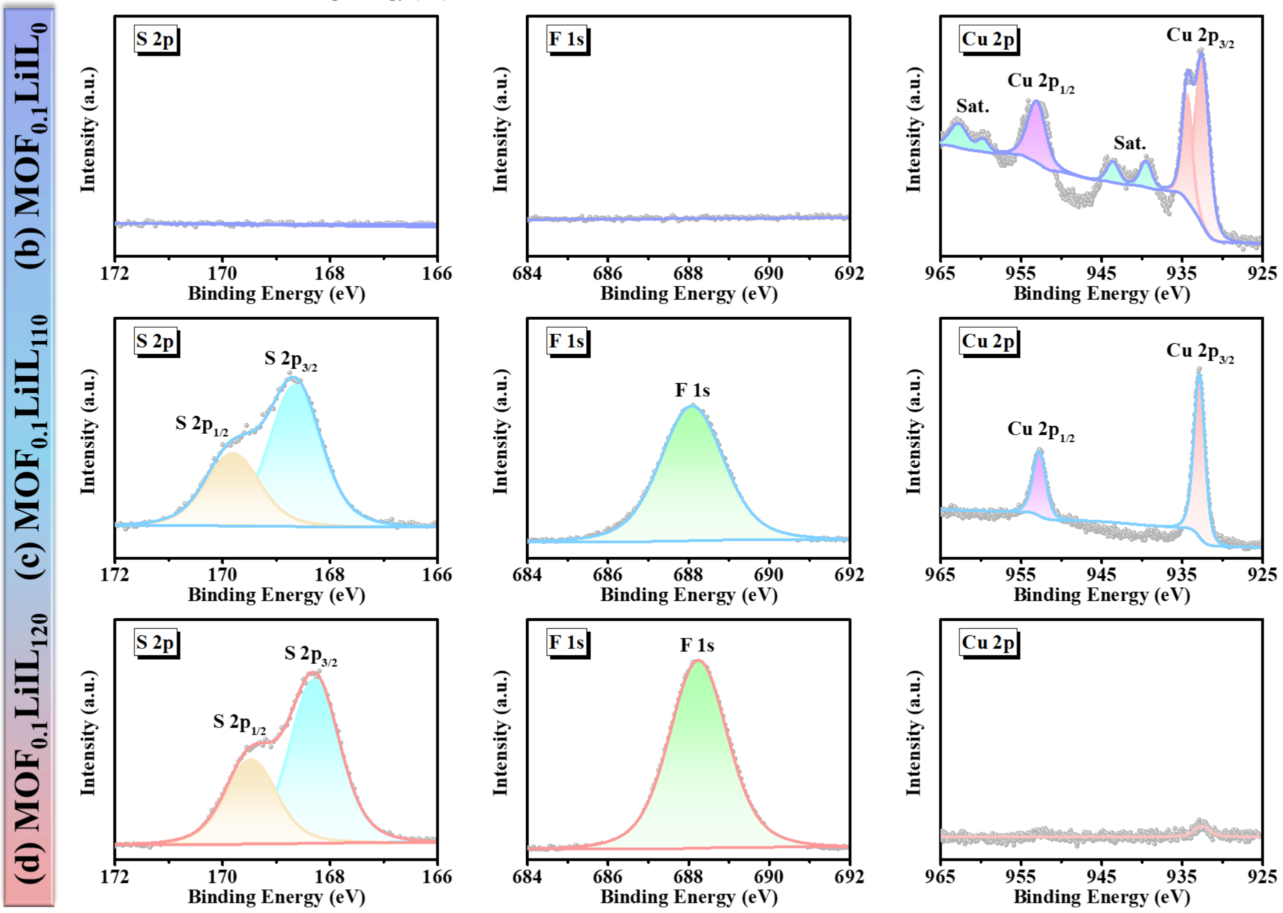

Fig. S3 (a-d) XPS spectra of $MOF_{0.1}LiIL_0$, $MOF_{0.1}LiIL_{110}$ and $MOF_{0.1}LiIL_{120}$ composites

The diversity of chemical composition and pore structure endows MOFs a higher designability compared to conventional inorganic fillers. The ordered pore channels of MOFs help to promote the uniformity of Li deposition and show great potential in improving the inhibition effect of the polymer matrix on Li dendrites [S1]. Unfortunately, CSEs simply packed with MOFs offer very limited improvements in ionic conductivity for practical applications [S2]. Ionic liquid-modified MOFs have been used as a novel ion-conductive filler for the preparation of CSEs with high ionic conductivity [S3]. In this case, the porous MOFs host provides a stable 3D open solid framework and the LiIL guest is encapsulated in the MOFs pore channels as a Li conductor, where the LiIL loses the free mobility of its primitive liquid but retains the high ionic conductivity properties [S4]. Therefore, the high LiIL content in MOFs is beneficial to enhance the ionic conductivity of CSEs, and we further confirm the optimal loading of LiIL. As shown in **Fig. S2a-f**, when the LiIL content was less than 110 μL, the MOFs still exhibited a polyhedral structure and the powder showed a dry state, which could eliminate

the risk of liquid leakage [S5]. However, when the LiIL content was 120 μL, the surface of MOFs was completely covered, the liquid overflowed and the powder showed a gel-like appearance, which indicated that the excess liquid LiIL could not be absorbed by the MOFs host. The XRD (**Fig. S2g**) shows that the intensity of MOFs characteristic peaks decreases with the increase of LiIL loading, but the peak position did not change, indicating that the MOFs hosts were chemically stable to LiIL. As the MOFs were filled with LiIL, their specific surface area (**Fig. S2i**) decreased from the initial 831.9 to 0.017 m^2 g^{-1}. To confirm that LiIL fills the pores of MOFs, XPS analysis was performed. It is worth noting that XPS can usually only detect the surface properties of the material. As shown in **Fig. S3**, the atomic percentages of S, F, Cu, and C in the MOFs are quantified by XPS survey. Since S and F are mainly derived from LiIL, S and F are almost absent in $MOF_{0.1}LiIL_0$. Diffraction peaks of S, F, and Cu are observed in $MOF_{0.1}LiIL_{110}$ when the IL content is 110 μL. The diffraction peaks of Cu in $MOF_{0.1}LiIL_{120}$ are almost undetectable when excess IL is added, providing strong evidence for the fact that LiIL are encapsulated in MOF pores [S6]. EDS mapping (**Fig. S4**) further illustrates that Cu, C, S and F are uniformly distributed in the MOFs framework.

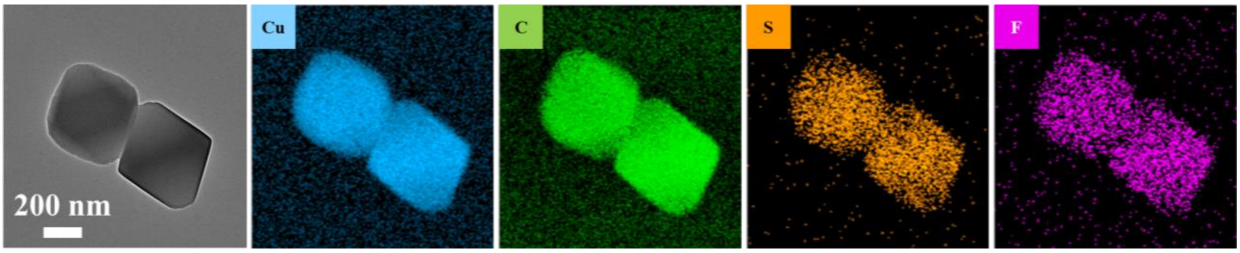

Fig. S4 TEM images and corresponding EDS mapping of $MOF_{0.1}LiIL_{110}$ composites

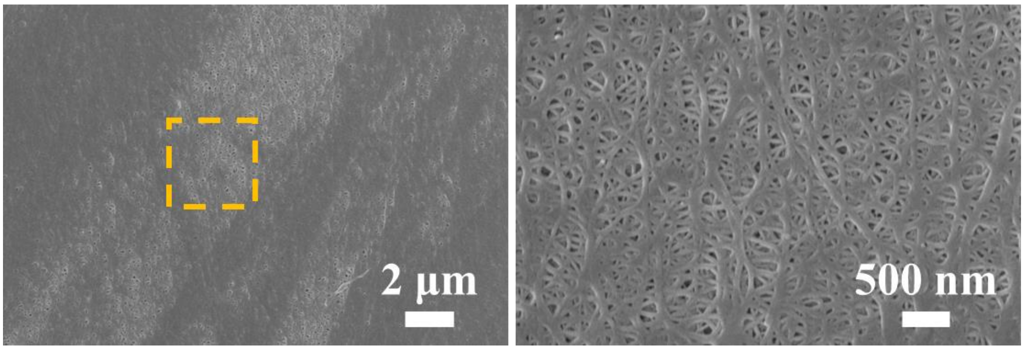

Fig. S5 SEM images of PE separator

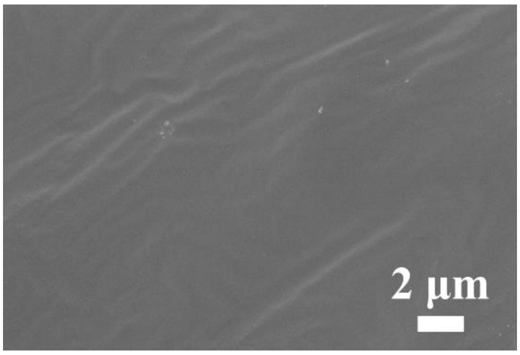

Fig. S6 SEM images of PEO

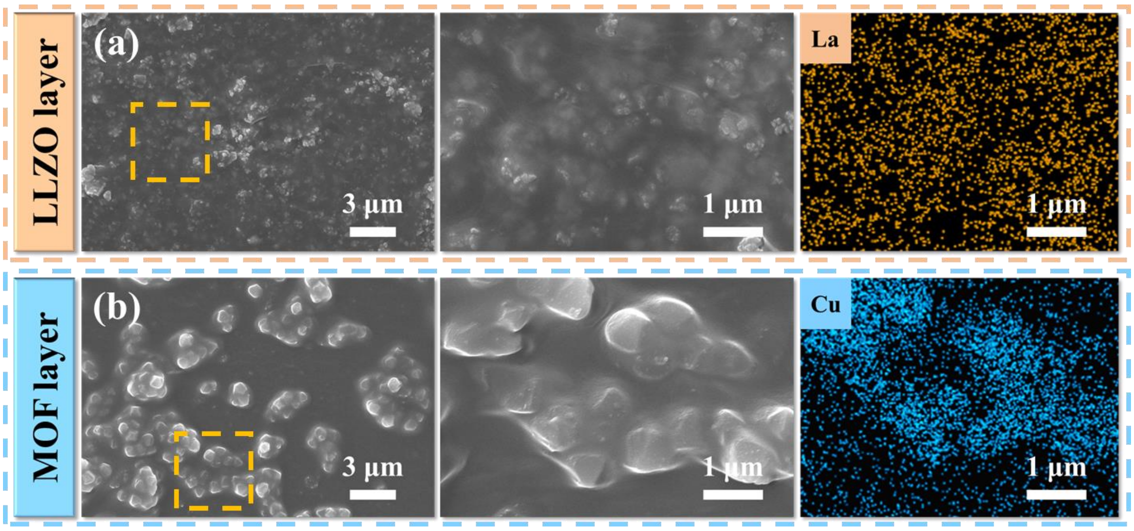

Fig. S7 SEM images of (**a**) LLZO layer and (**b**) MOF layer with corresponding EDS mapping

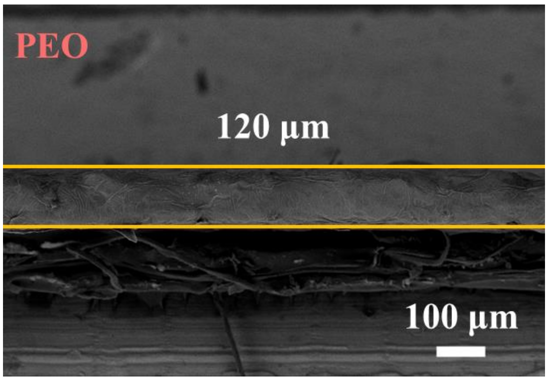

Fig. S8 Cross-sectional SEM image of PEO electrolyte

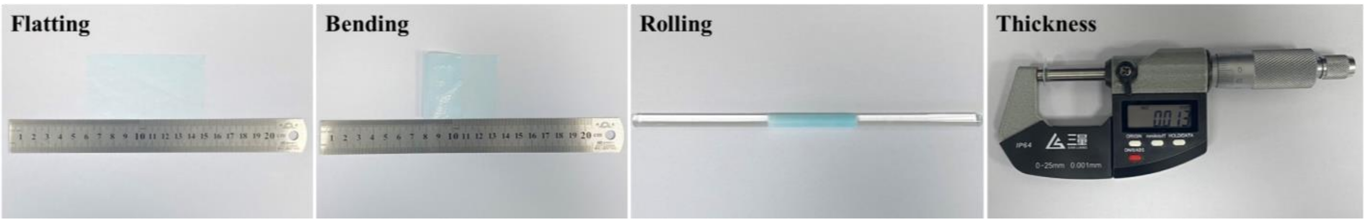

Fig. S9 Optical images of PLM in different states

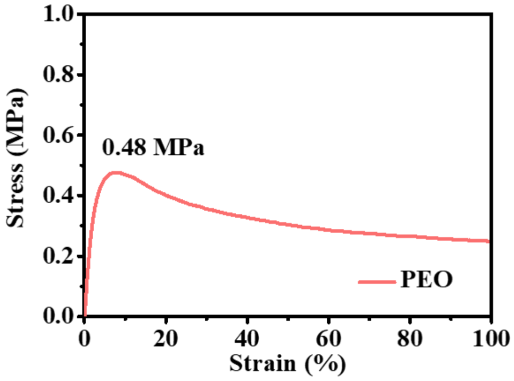

Fig. S10 Stress-strain curve of PEO

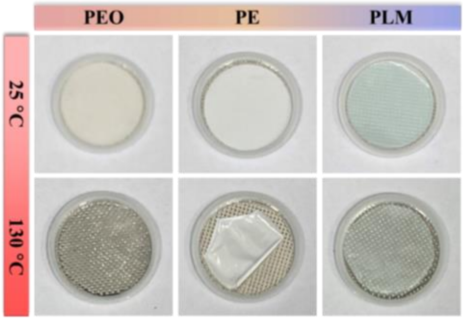

Fig. S11 The evolution of the electrolyte state with temperature

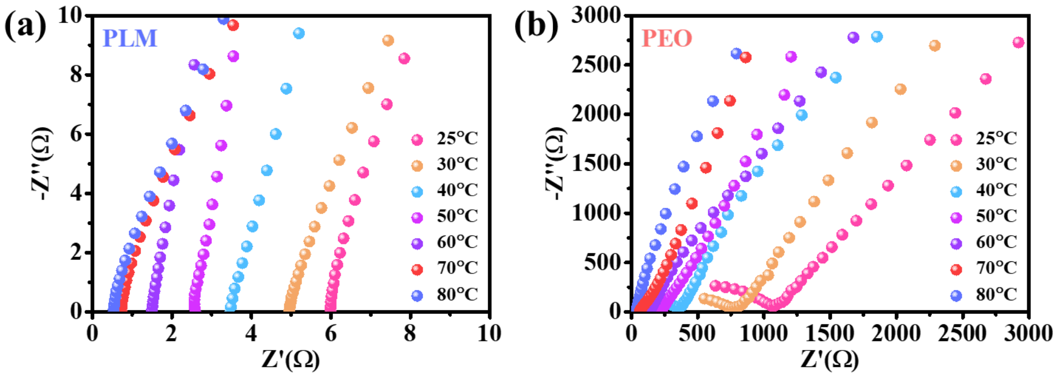

Fig. S12 EIS curves of (**a**) SS/PLM/SS and (**b**) SS/PEO/SS

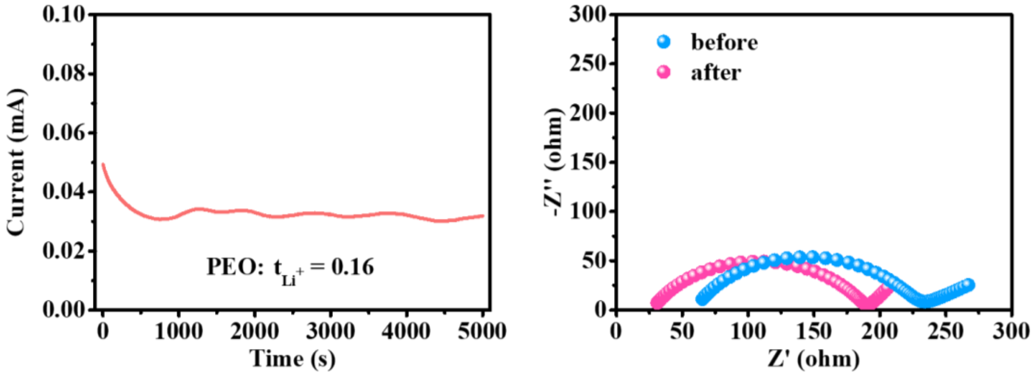

Fig. S13 Li^+ transference number of PEO

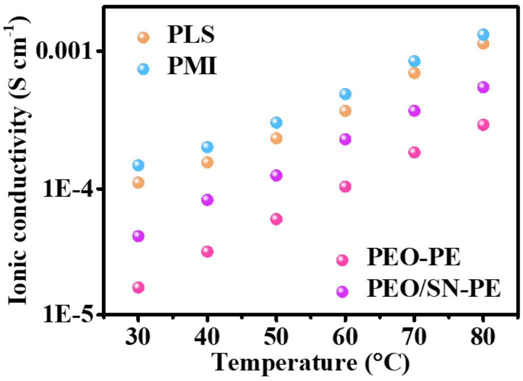

Fig. S14 Ion conductivity of PLS, PMI, PEO-PE and PEO/SN-PE

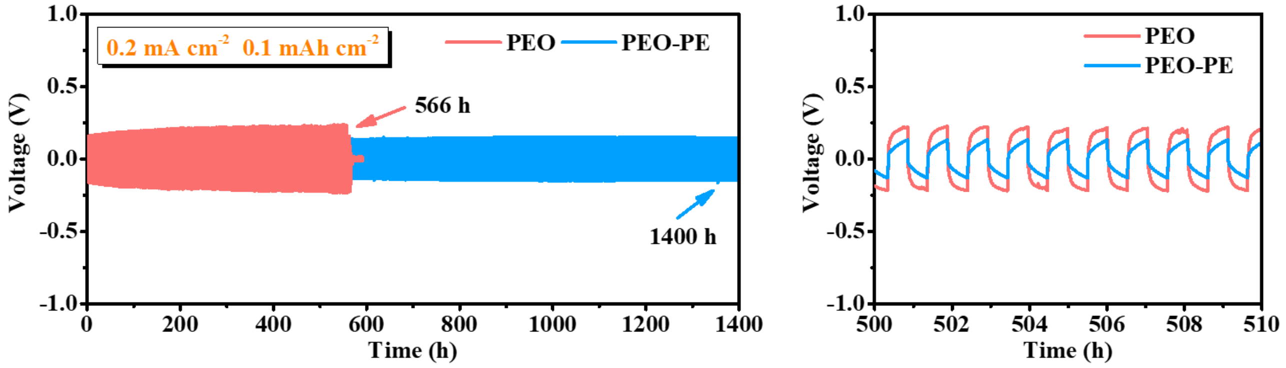

Fig. S15 Li plating/stripping curves of PEO and PEO-PE at 0.2 mA cm^{-2}/0.1 mAh cm^{-2}

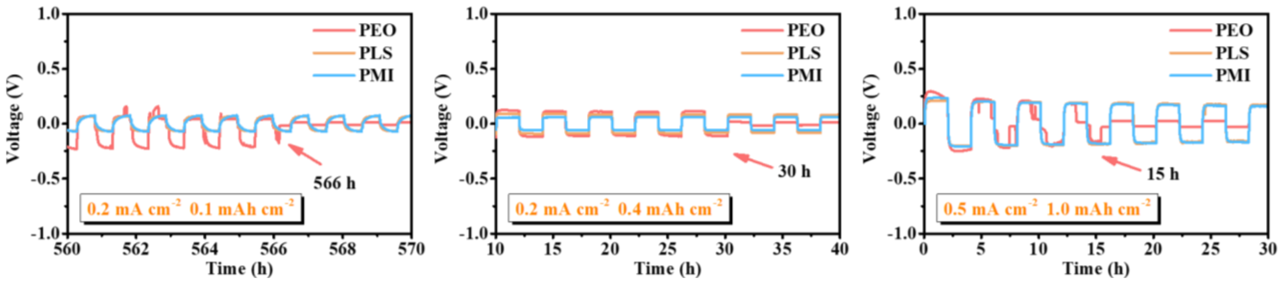

Fig. S16 Amplified Li plating/stripping curves when PEO electrolyte is short-circuited

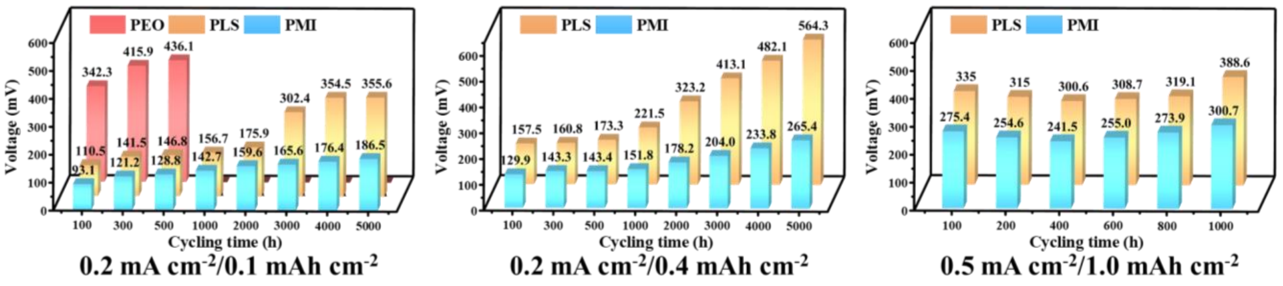

Fig. S17 Polarization potential of Li-symmetric batteries at different times

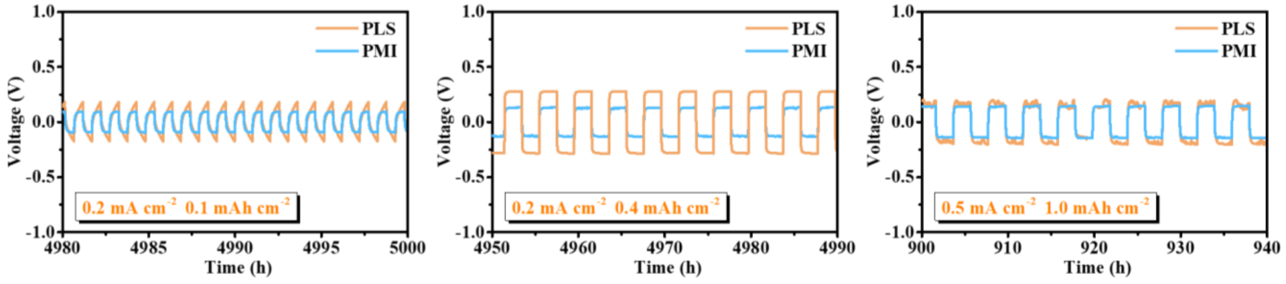

Fig. S18 Comparison of Li plating/stripping curves of PMI and PLS

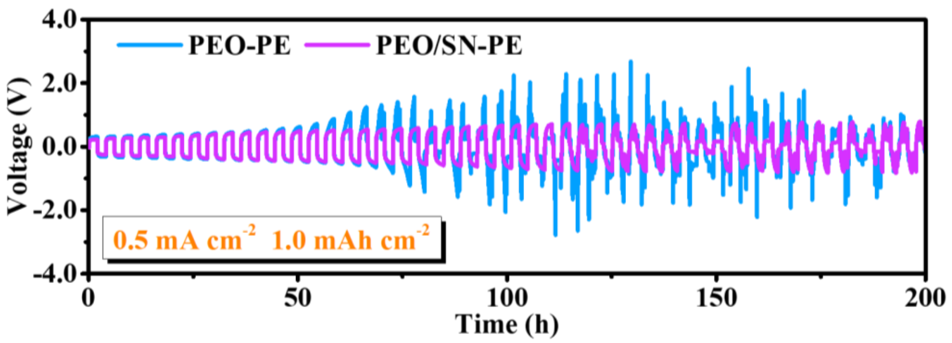

Fig. S19 Li plating/stripping curves of PEO-PE and PEO/SN-PE at 0.5 mA cm^{-2}/1.0 mAh cm^{-2}

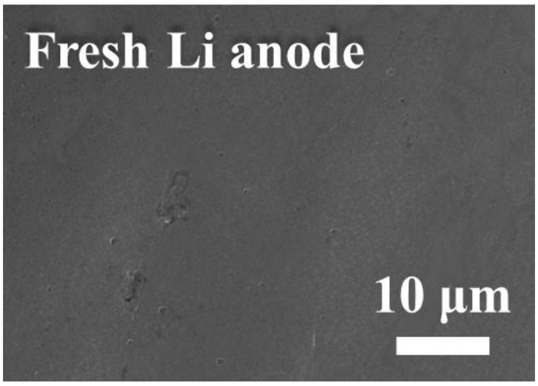

Fig. S20 Surface morphology of fresh Li anode before cycling

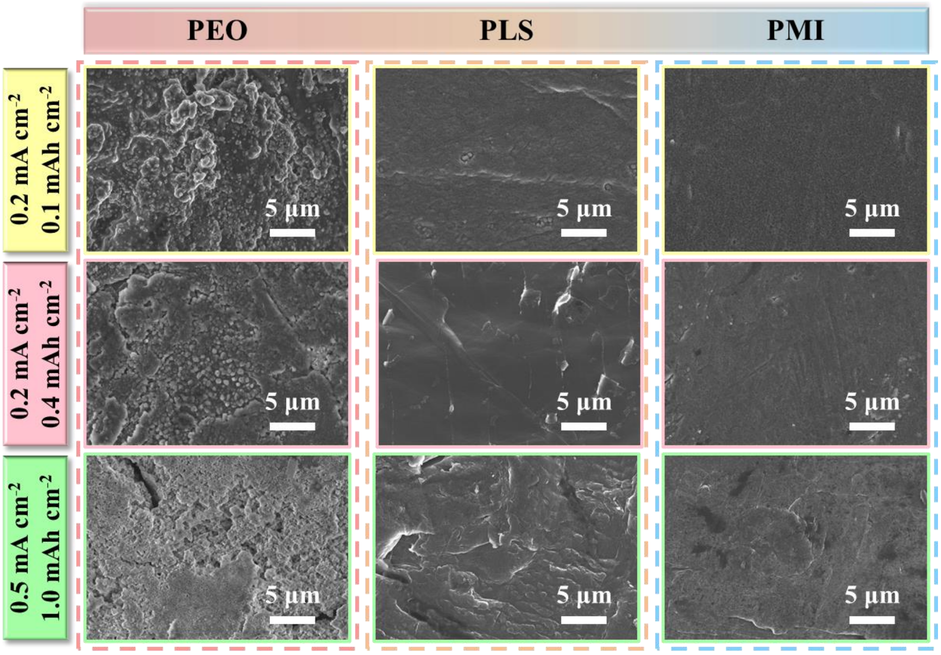

Fig. S21 Surface morphology of Li anode after 400 h of Li-symmetric batteries cycling (Li/PEO/Li batteries are based on short circuit time)

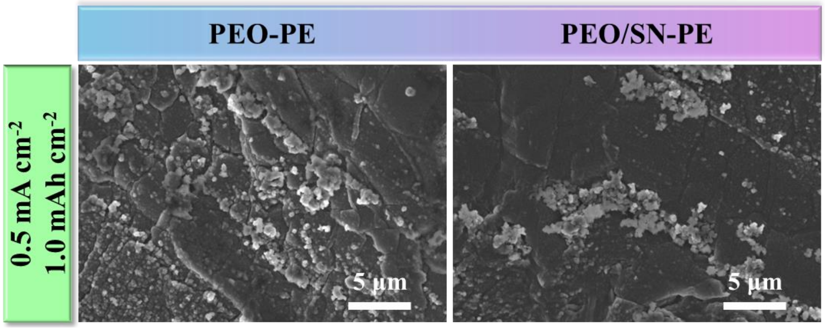

Fig. S22 Surface morphology of Li anode after 200 h

The pristine Li anode has a flat surface (**Fig. S20**). As shown in **Fig. S21**, in the Li/PEO/Li batteries, there is a large amount of dead Li on the Li anode surface, which gradually pulverizes with increasing Li deposition capacity. Although PLS has a smoother Li anode surface at low deposition capacity, due to the corrosion of Li metal by SN, uneven dot-like deposition on the Li anode surface is observed at increased deposition capacity (0.4 mAh cm^{-2}, 1.0 mAh cm^{-2}). As a result of the joint promotion of the PE separator and the MOF layer, a denser Li anode surfaces without Li dendrites have been observed in the Li/PMI/Li batteries. For comparison, the Li plating/stripping curves of Li symmetric batteries with PEO-PE and PEO/SN-PE electrolytes at 0.5 mA cm^{-2}/1.0 mAh cm^{-2} were also verified (**Fig. S19**). Although no short-circuiting occurred in the Li symmetric batteries, the overpotentials of both batteries gradually increased with time. Due to the severe polarization phenomenon, a large number of irregular deposits existed on the surface of the Li anode from the symmetric batteries (**Fig. S22**).

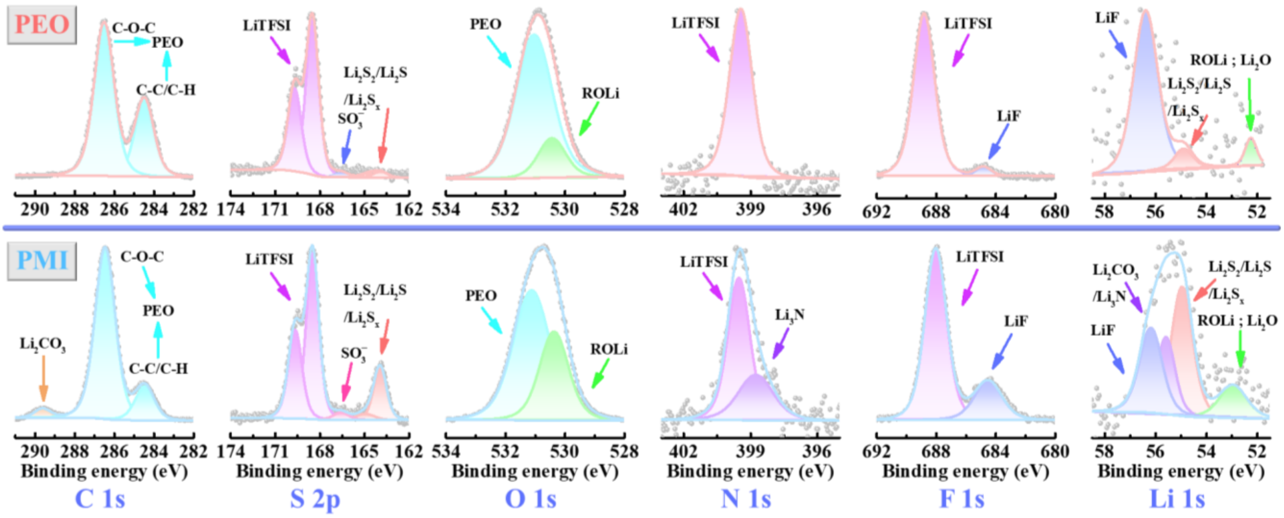

Fig. S23 XPS spectra of PEO and PMI after cycling

Simulation of Li dendrite evolution

From the physical point of view, Li^+ move towards the electrode surface because of the overpotential divergence between the anode and cathode. As a result, Li^+ are reduced to lithium atoms and deposits on the Li foil surface, inducing the formation and growth of Li dendrites. In this situation, electrochemistry and mechanics are closely coupled within the confined space of the electrolyte/Li interface: the volume change of Li dendrites compresses the electrolyte and the opposite stress retard the electrochemical reaction rate, thus reshaping the Li dendrites. To simulate the complicated morphological evolution of Li dendrites regulated by the mechano-electrochemical phenomenon, a Phase-field model (PFM) has been developed. For simplicity, two reasonable assumptions are made: First, the PE skeleton are configured as uniformly aligned filaments perpendicular to the anode surface. Second, the impact of lithium dendrites on the PE skeleton are ignored due to the large gap of the Young's modulus between the PE skeleton and PEO electrolyte. The time-dependent evolution of Li electroplating is expressed by the following equations:

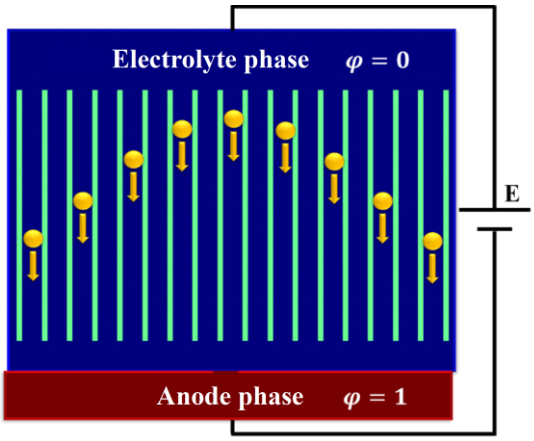

Fig. S24 The schematic illustration of the computational domain

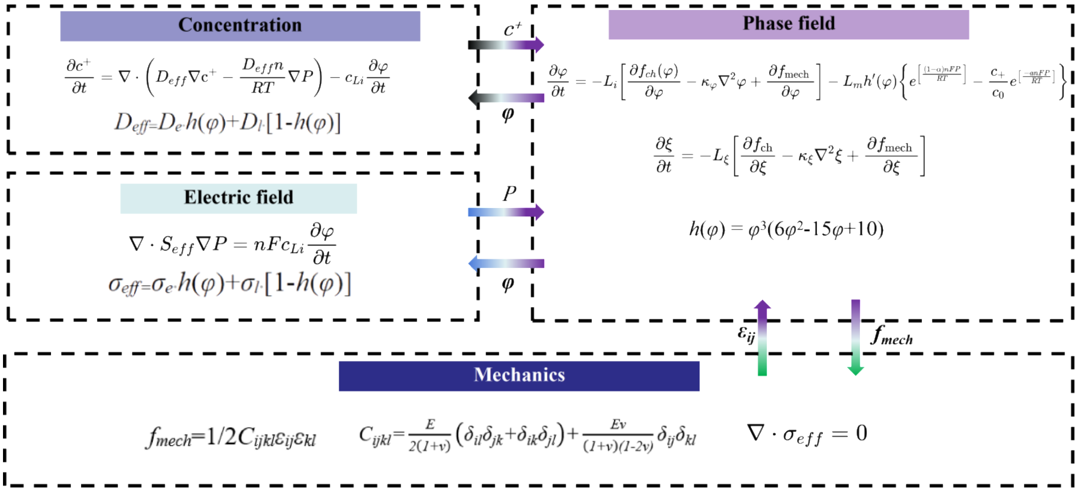

Fig. S25 The governing equations based on the phase-field model

Simulation of Li⁺ concentration, electric potential, anion concentration distribution

The transport of Li^+ stimulated by electric fields were imitated based on Electrostatic and Transport of Diluted Species modules combined in COMSOL Multiphysics. The physical model is governed by Nernst-Planck-Poisson equations:

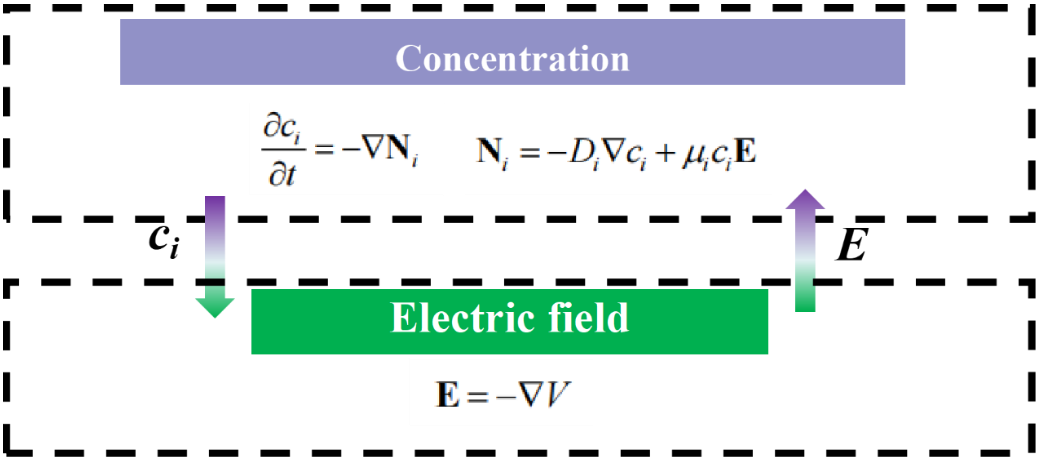

Fig. S26 The Nernst-Planck-Poisson equations

where V is the overpotential, E is the electric field, D_i is the diffusion coefficient of species i, c_i is the concentration of species i, μ_i is the ionic mobility of species i, and Ni is the flux vector of species i. As the driving force for Li^+ transport, the overpotential difference between the anode and cathode was set as 0.01 V. The Li^+ flux on the boundary, which resulted from the electrochemical reaction, was set as 0.01 mol m^{-2} s^{-1}. The diffusion coefficient is derived from the ionic conductivity based on Nernst-Einstein equation.

For the comparison of PEO and PEO/MOFs, the computational domain was configured as a rectangle with the size of 100 × 20 μm^2. According to the Li^+ transference number, anions were divided into two sets: one corresponds to the free anions with the normal diffusion coefficient, the other corresponds to the immobilized anions with the diffusion coefficient of 0.

For the comparison of PEO and PLM, the computational domain was configured as a rectangle with the size of 20 × 5.6 μm^2, which is in accordance with the SEM results. To highlight the functionality of the PEO and LLZO/MOF coatings, the PE is equivalent to a thin layer in the PLM case. Moreover, the diffusion coefficient and Li^+ transference number in each layer were taken into account via the aforementioned method.

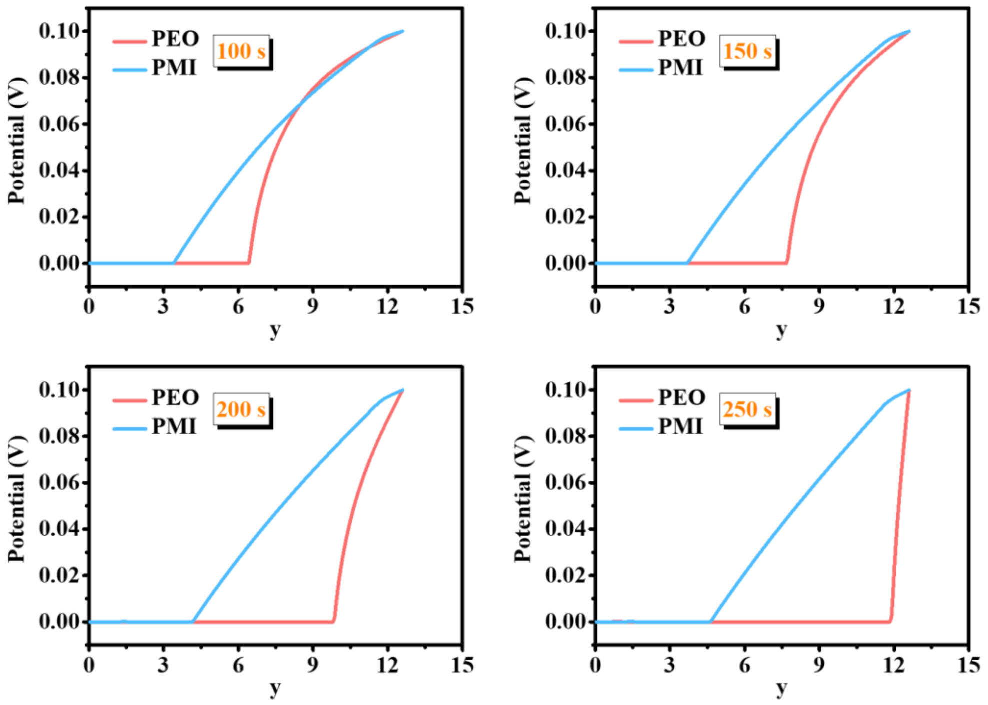

Fig. S27 The potential distribution along the central axis of the electric field

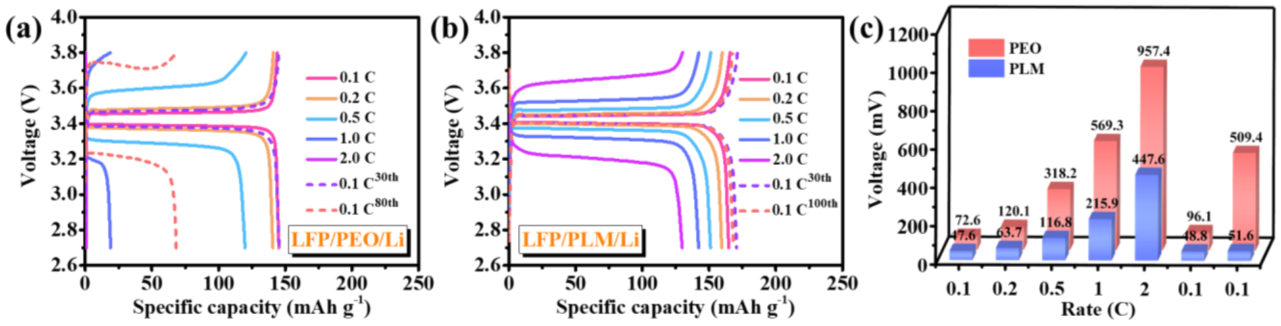

Fig. S28 Galvanostatic charge/discharg curves of (a) LFP/PEO/Li and (b) LFP/PLM/Li at 60 °C. (c) Comparison of polarization potentials at different rates (60 °C).

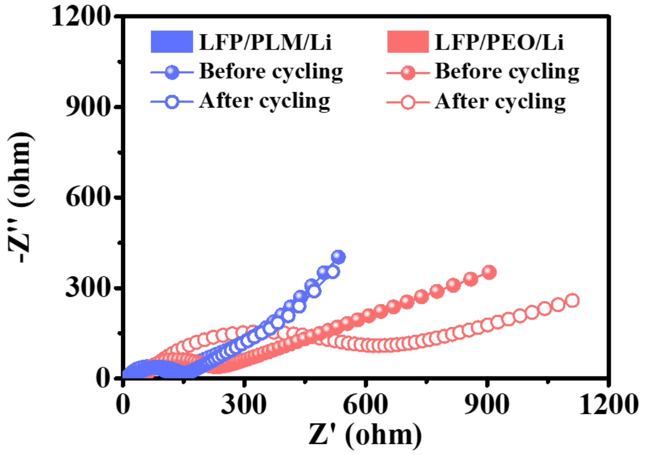

Fig. S29 EIS curves of LFP/CSEs/Li batteries before and after rate cycling at 60 °C

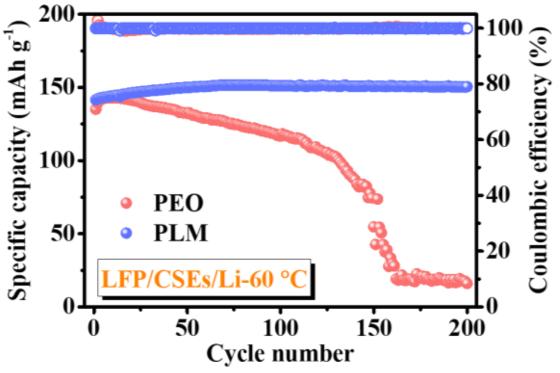

Fig. S30 Cycling performance of LFP/CSEs/Li batteries at 0.5 C (60 °C)

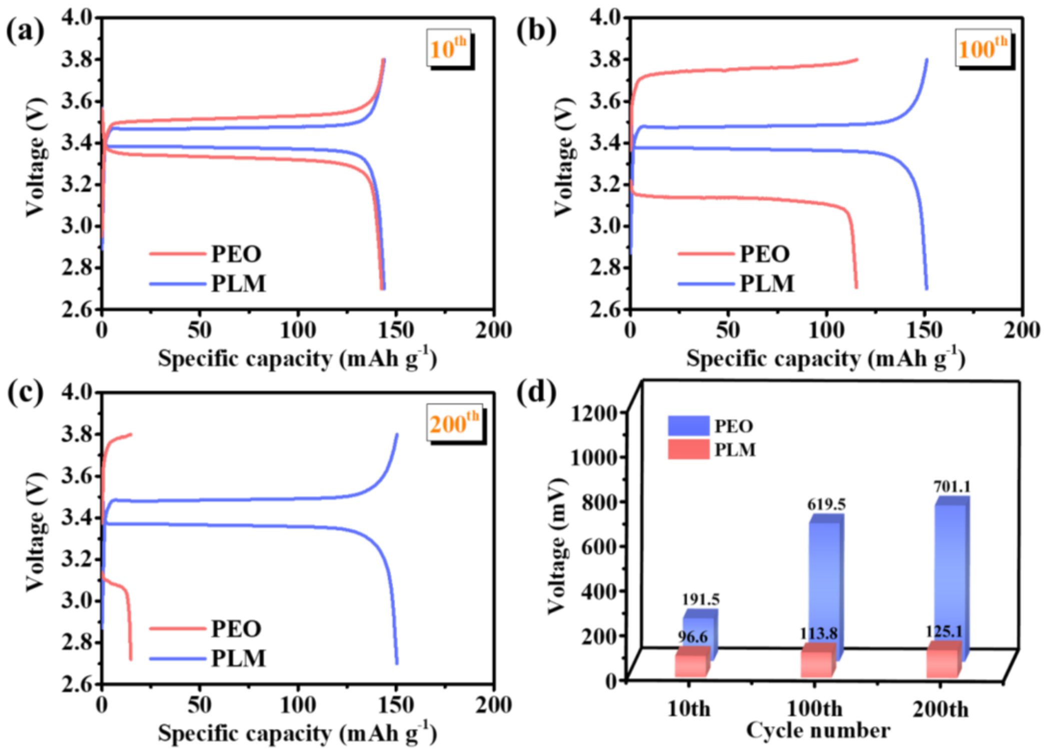

Fig. S31 Galvanostatic charge/discharg curves of LFP/CSEs/Li at different cycles (0.5 C, 60 °C). (**a**) 10th, (**b**) 100th, (**c**) 200th. (**d**) Comparison of polarization potentials at different cycles

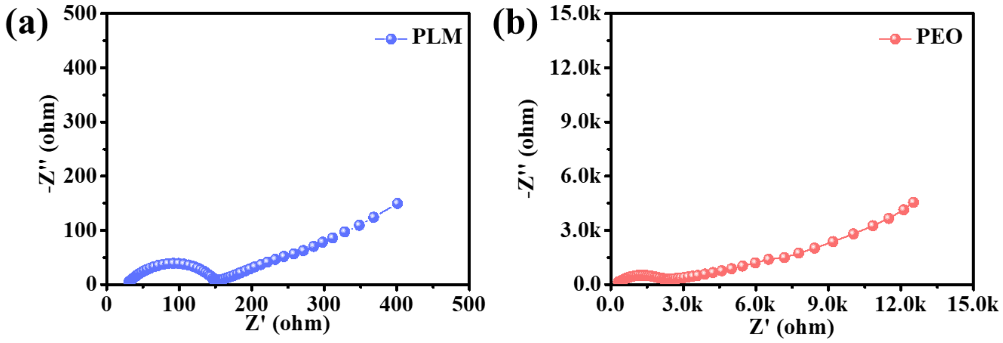

Fig. S32 EIS curves of LFP/CSEs/Li batteries after 200 cycles at 0.5 C (60 °C). (**a**) LFP/PLM/Li and (**b**) LFP/PEO/Li

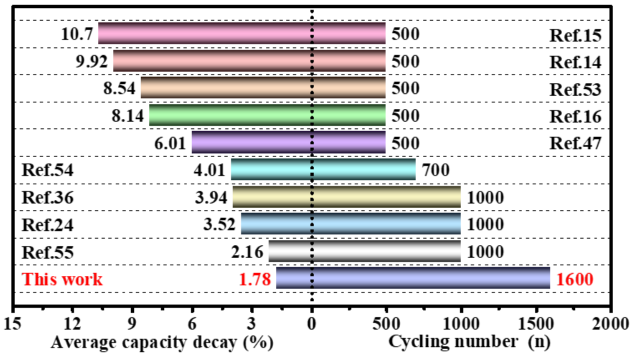

Fig. S33 Relationship between average capacity decay and cycle life, and comparison with previously reported LFP-based batteries with ultrathin electrolytes

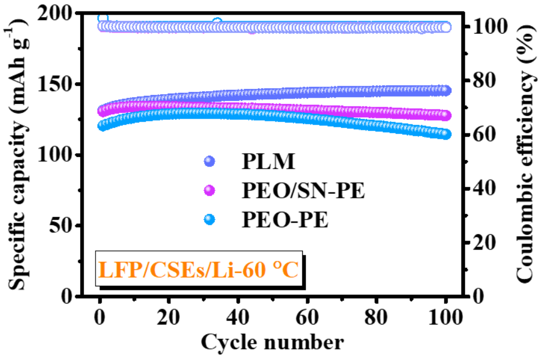

Fig. S34 Cycling performance of LFP/CSEs/Li batteries at 1.0 C (60 °C)

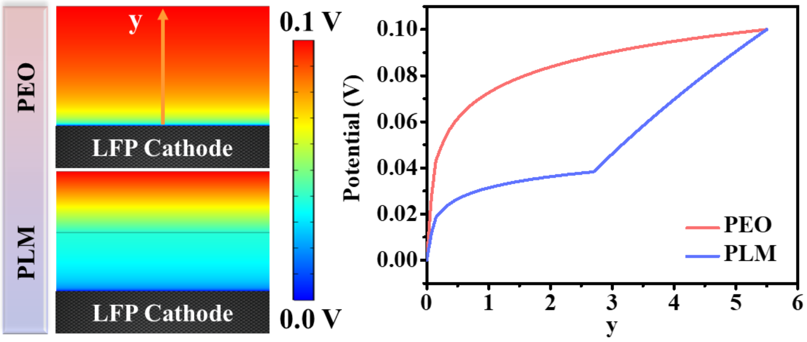

Fig. S35 Distribution of electric potential in SSLMBs with PEO and PMI electrolyte

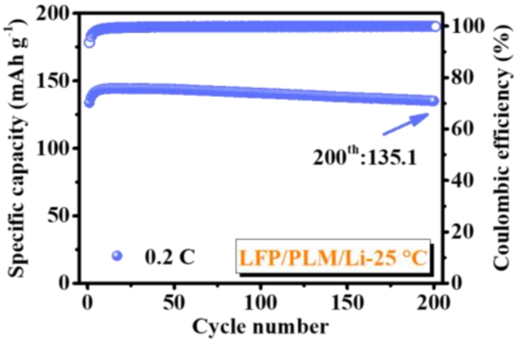

Fig. S36 Cycling performance of LFP/PLM/Li at 0.2 C (25 °C)

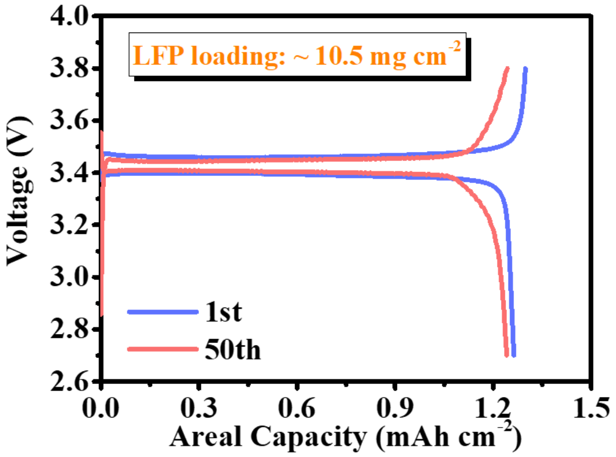

Fig. S37 Galvanostatic charge/discharg curves of LFP/PLM/Li batteries with LFP loading 10.5 mg cm^{-2} at 0.1 C (25 °C)

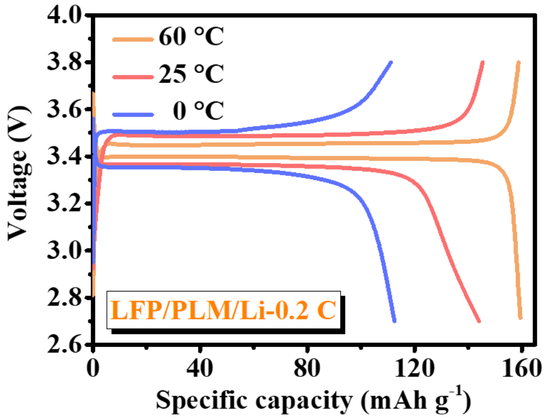

Fig. S38 Galvanostatic charge/discharg curves of LFP/PLM/Li batteries with different temperature (60 °C, 25 °C, 0 °C) at 0.2 C

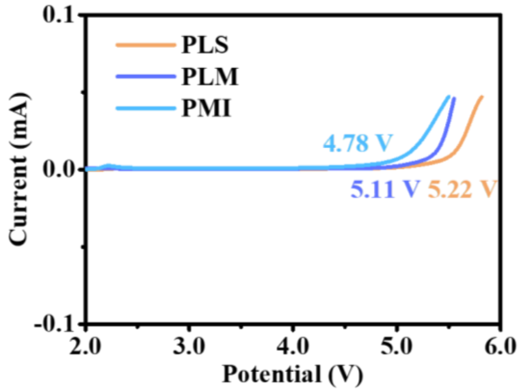

Fig. S39 LSV curves of PLS, PLM and PMI

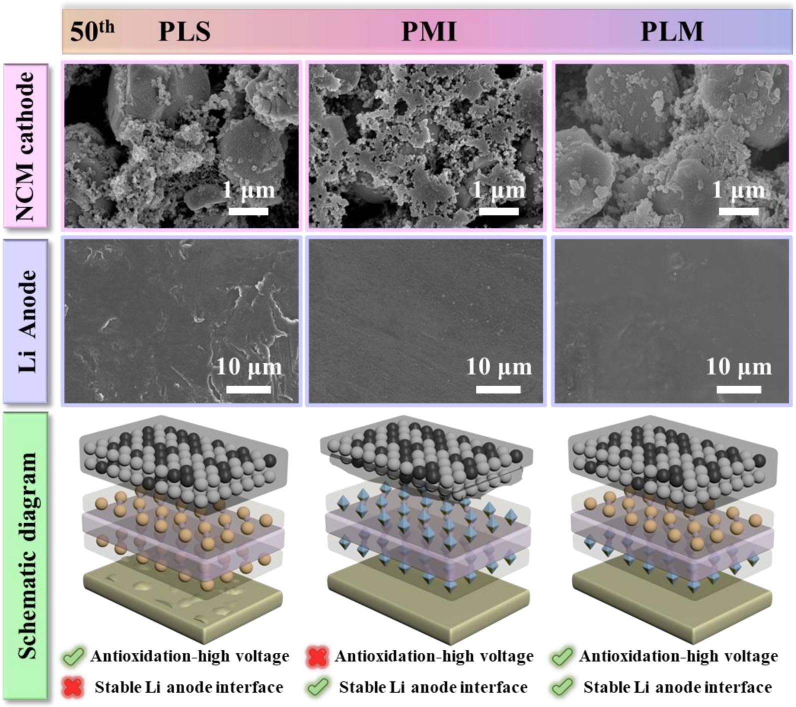

Fig. S40 Surface morphologies of the NCM cathode and Li anode after 50 cycles at 0.2 C (NCM811/CSEs/Li)

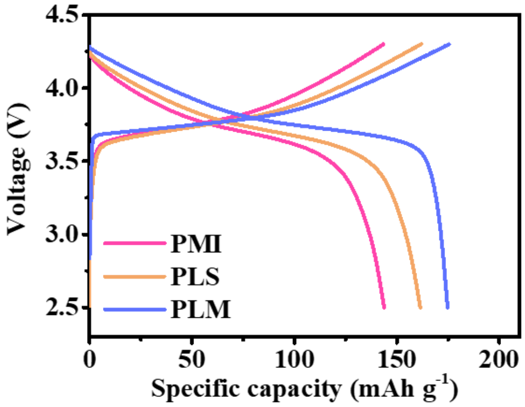

Fig. S41 Galvanostatic charge/discharg curves of NCM811/CSEs/Li at 50th (0.2 C)

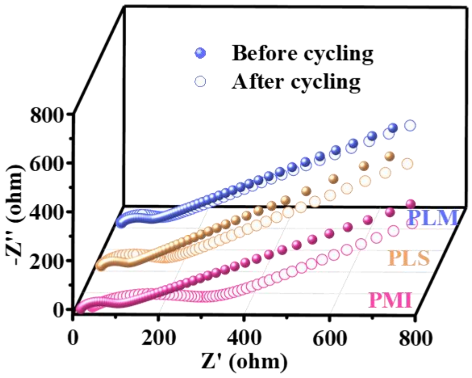

Fig. S42 EIS curves of NCM811/CSEs/Li before and after 50 cycles

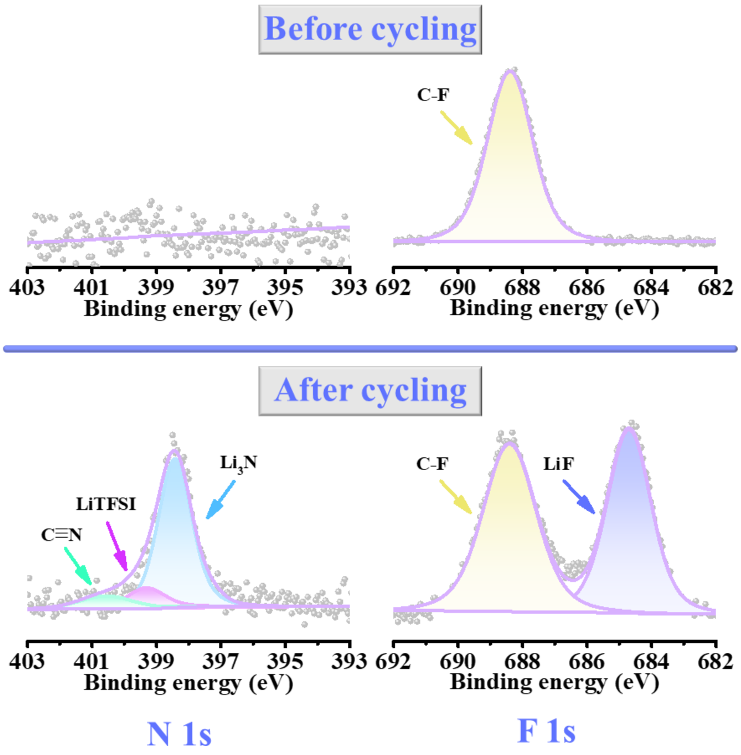

Fig. S43 XPS analysis of NCM anode before and after 50 cycles of NCM811/PLM/Li battery (0.2 C)

Table S1 The important parameters of governing equations

**Parameter**	**Symbol**	**Real value**
Interfacial mobility	L_i	2.510^{-6} m^3 J^{-1} s^{-1}
Barrier height	W	3.7510^5 J m^{-3}
Reaction coefficient	L_m	1 s^{-1}
Gradient energy coefficient	κ_0	2.510^{-5} J m^{-1}
Diffusivity in electrode	D_e	7.510^{-13} m^2 s^{-1}
Diffusivity in electrolyte	D_l	2.510^{-10} m^2 s^{-1}
Conductivity in electrode	σ_e	10^7 S m^{-1}
Symmetrical factors	α, β	0.5
Elastic modulus of Li metal	E_m	4.9 GPa
Elastic modulus of PEO	E_e	12.6 MPa
Temperature	T	300 K

Table S2 Comparison of the areal capacity of LFP-based SSLMBs with relevant CSEs in recent publications

Electrolyte	LFP loading (mg cm^{-2})	Areal capacity (mAh cm^{-2})	Thickness (μm)	Tempreature (°C)
CPDOL-SPE [S7]	1.3	0.21	16	60
PLCPB [S8]	2.5	0.4	56	60
NCSE-VA [S9]	3	0.48	46.2	60
3D-SPE [S10]	5	0.58	200	25
Li-MC [S11]	6	0.95	30	25
PAI-SPE [S12]	6.9	0.97	100	45
PLP-HP [S13]	7.64	0.95	14.5	60
PALE [S14]	8.39	0.95	67	25
FPG [S15]	13.0	1.61	25	45
FG-SPE [S16]	15.6	1.54	20	25
**This work**	**10.5**	**1.26**	**12.6**	**25**

Table S3 Cycling performance comparison of NCM-based SSLMBs with recently publications

Electrolyte	Thickness (μm)	Cathode	Cycling number (n)	Specific capacity (mAh g^{-1})	Capacity retention (%)	Average capacity decay (%)	Temperature (°C)
MOF@PAN/PEO/IL CPE [S6]	82.8	NCM811	150	115.4	74	27	25
Si@LATP/PVDF/PVC [S17]	50	NCM532	100	124.6	86.4	19.6	25
3D-LLZT-CSE [S18]	95	NCM811	230	118.8	79.4	13.4	30
PVLN [S19]	100	NCM811	500	136.4	80.8	6.5	25
PVBL [S20]	67	NCM811	1000	118.1	70	5.06	25
**This work**	**12.6**	**NCM811**	**500**	**131.3**	**81.3**	**6.0**	**25**

Table S4 Calculation of the gravimetric/volumetric energy density of Li/PLM/NCM811 pouch cell

Component of pouch cell	Parameter		Real value
Cathode (NCM811)	Active material loading (mg cm^{-2})		20.3
	NCM811 ratio (%)		94.5
	Thickness (μm)		90
	Areal capacity (mAh cm^{-2})		3.19
PLM Electrolyte	Thickness (μm)		12.6
	Areal density (mg cm^{-2})		1.69
Anode (Li)	Thickness (μm)		40
	Areal density (mg cm^{-2})		2.14
Current collector (Al)	Thickness (μm)		14
	Areal density (mg cm^{-2})		3.78
Liquid electrolyte	Areal density	μL cm^{-2}	5
		mg cm^{-2}	6.1
Pouch cell	Total mass (mg cm^{-2})		35.19
	Total thickness (μm)		156.6
	Average discharge voltage (V)		3.795
	Gravimetric energy density (Wh kg^{-1})		344.0
	Volumetric energy density (Wh L^{-1})		773.1

Supplementary References

[S1] S. Liu, W. Liu, D. Ba, Y. Zhao, Y. Ye et al., Filler-integrated composite polymer electrolyte for solid-state lithium batteries. Adv. Mater. **35**, 2110423 (2023). https://doi.org/10.1002/adma.202110423

[S2] C.-C. Sun, A. Yusuf, S.-W. Li, X.-L. Qi, Y. Ma et al., Metal organic frameworks enabled rational design of multifunctional peo-based solid polymer electrolytes. Chem. Eng. J. **414**, 128702 (2021). https://doi.org/10.1016/j.cej.2021.128702

[S3] R. Zhao, Y. Wu, Z. Liang, L. Gao, W. Xia et al., Metal–organic frameworks for solid-state electrolytes. Energy Environ. Sci. **13**, 2386-2403 (2020). https://doi.org/10.1039/D0EE00153H

[S4] Z. Wang, R. Tan, H. Wang, L. Yang, J. Hu et al., A metal–organic-framework-based electrolyte with nanowetted interfaces for high-energy-density solid-state lithium battery. Adv. Mater. **30**, 1704436 (2018). https://doi.org/10.1002/adma.201704436

[S5] Z. Wang, Z. Wang, L. Yang, H. Wang, Y. Song et al., Boosting interfacial Li^+ transport with

a mof-based ionic conductor for solid-state batteries. Nano Energy **49**, 580-587 (2018). https://doi.org/10.1016/j.nanoen.2018.04.076

[S6] X.-L. Zhang, F.-Y. Shen, X. Long, S. Zheng, Z. Ruan et al., Fast Li^+ transport and superior interfacial chemistry within composite polymer electrolyte enables ultra-long cycling solid-state li-metal batteries. Energy Stor. Mater. **52**, 201-209 (2022). https://doi.org/10.1016/j.ensm.2022.07.045

[S7] H. Xu, J. Zhang, H. Zhang, J. Long, L. Xu et al., In situ topological interphases boosting stable solid-state lithium metal batteries. Adv. Energy Mater. **13**, 2204411 (2023). https://doi.org/10.1002/aenm.202204411

[S8] J. Song, Y. Xu, Y. Zhou, P. Wang, H. Feng et al., Cellulose-assisted vertically heterostructured peo-based solid electrolytes mitigating Li-succinonitrile corrosion for lithium metal batteries. ACS Appl. Mater. Interfaces **15**, 20897-20908 (2023). https://doi.org/10.1021/acsami.2c22562

[S9] Y. Nie, T. Yang, D. Luo, Y. Liu, Q. Ma et al., Tailoring vertically aligned inorganic-polymer nanocomposites with abundant lewis acid sites for ultra-stable solid-state lithium metal batteries. Adv. Energy Mater. **13**, 2204218 (2023). https://doi.org/10.1002/aenm.202204218

[S10] Y. He, S. Chen, L. Nie, Z. Sun, X. Wu et al., Stereolithography three-dimensional printing solid polymer electrolytes for all-solid-state lithium metal batteries. Nano Lett. **20**, 7136-7143 (2020). https://doi.org/10.1021/acs.nanolett.0c02457

[S11] Y. Zheng, N. Yang, R. Gao, Z. Li, H. Dou et al., "Tree-trunk" design for flexible quasi-solid-state electrolytes with hierarchical ion-channels enabling ultralong-life lithium-metal batteries. Adv. Mater. **34**, 2203417 (2022). https://doi.org/10.1002/adma.202203417

[S12] D. Zhang, Z. Liu, Y. Wu, S. Ji, Z. Yuan et al., In situ construction a stable protective layer in polymer electrolyte for ultralong lifespan solid-state lithium metal batteries. Adv. Sci. **9**, 2104277 (2022). https://doi.org/10.1002/advs.202104277

[S13] Q. Liang, L. Chen, J. Tang, X. Liu, J. Liu et al., Large-scale preparation of ultrathin composite polymer electrolytes with excellent mechanical properties and high thermal stability for solid-state lithium-metal batteries. Energy Stor. Mater. **55**, 847-856 (2023). https://doi.org/10.1016/j.ensm.2022.12.039

[S14] S. Chai, Z. Chang, Y. Zhong, Q. He, Y. Wang et al., Regulation of interphase layer by flexible quasi-solid block polymer electrolyte to achieve highly stable lithium metal batteries. Adv. Funct. Mater. **33**, 2300425 (2023). https://doi.org/10.1002/adfm.202300425

[S15] Y. Lin, M. Wu, J. Sun, L. Zhang, Q. Jian et al., A high-capacity, long-cycling all-solid-state lithium battery enabled by integrated cathode/ultrathin solid electrolyte. Adv. Energy Mater. **11**, 2101612 (2021). https://doi.org/10.1002/aenm.202101612

[S16] J. Liu, J. Zhou, M. Wang, C. Niu, T. Qian et al., A functional-gradient-structured ultrahigh modulus solid polymer electrolyte for all-solid-state lithium metal batteries. J. Mater. Chem. A. **7**, 24477-24485 (2019). https://doi.org/10.1039/C9TA07876B

[S17] Y. Jin, X. Zong, X. Zhang, Z. Jia, H. Xie et al., Constructing 3D Li^+-percolated transport network in composite polymer electrolytes for rechargeable quasi-solid-state lithium batteries. Energy Stor. Mater. **49**, 433-444 (2022). https://doi.org/10.1016/j.ensm.2022.04.035

[S18] A.-G. Nguyen, R. Verma, G.-C. Song, J. Kim, C.-J. Park. In situ polymerization on a 3D eramic framework of composite solid electrolytes for room-temperature solid-state batteries. Adv. Sci. **10**, 2207744 (2023). https://doi.org/10.1002/advs.202207744

[S19] K. Yang, L. Chen, J. Ma, C. Lai, Y. Huang et al., Stable interface chemistry and multiple ion transport of composite electrolyte contribute to ultra-long cycling solid-state $LiNi_{0.8}Co_{0.1}Mn_{0.1}O_2$/lithium metal batteries. Angew. Chem. Int. Ed. **60**, 24668-24675 (2021). https://doi.org/10.1002/anie.202110917

[S20] P. Shi, J. Ma, M. Liu, S. Guo, Y. Huang et al., A dielectric electrolyte composite with high lithium-ion conductivity for high-voltage solid-state lithium metal batteries. Nat. Nanotechnol. **18**, 602-610 (2023). https://doi.org/10.1038/s41565-023-01341-2
